# Across-cities transportable ^13^C hyperpolarization using UV-induced labile radicals

**DOI:** 10.1038/s41467-026-71466-0

**Published:** 2026-04-15

**Authors:** Andrea Capozzi, Magnus Karlsson, Yupeng Zhao, Jan Kilund, Esben Søvsø Szocska Hansen, Lotte Bonde Bertelsen, Christoffer Laustsen, Jan Henrik Ardenkjær-Larsen, Mathilde H. Lerche

**Affiliations:** 1 LIFMET, Department of Physics, EPFL, Lausanne, Switzerland; 2 HYPERMAG, Department of Health Technology, Technical University of Denmark, Kgs Lyngby, Denmark; 3 The MR Center, Department of Clinical Medicine, Aarhus University, Aarhus N, Denmark

**Keywords:** Solution-state NMR, Chemical physics

## Abstract

Hyperpolarized ^13^C Magnetic Resonance Spectroscopic Imaging (HP ^13^C-MRSI) has the potential to transform diagnostic radiology thanks to its unique ability to noninvasively detect a broad range of diseases entailing aberrant metabolism. However, clinical adoption has been hindered by the short lifetime of ^13^C-hyperpolarization and the resulting need for on-site polarizer near the MR scanner. In this work, we present a solution for long-lived transportable HP molecular contrast agents that uses dissolution Dynamic Nuclear Polarization (dDNP) combined with UV-induced labile radicals. This approach allows centralized pre-polarization and transport under hours-long storage T_1_ conditions. We validate this concept through the first across-cities HP ^13^C-MRSI experiments in vivo, injecting healthy female rats with both a perfusion/angiography ([1-^13^C]HP001) and a metabolic ([U-^13^C, d_7_]glucose) contrast agent. Our findings advance the feasibility of decentralized, scalable HP MRI workflows, removing the barrier of on-site infrastructure.

## Abstract (web summary)

A method using UV-generated radicals allows hyperpolarized ^13^C MRI agents to be produced centrally and transported for hours while preserving polarization, enabling in-vivo imaging off-site after long-distance transport.

## Introduction

In recent years, Magnetic Resonance Spectroscopic Imaging (MRSI) has emerged as one of the most promising molecular imaging techniques to detect biochemical changes in vivo^1–3^. By nature, MRSI has the advantage of being ionizing radiation-free and of having a spectroscopic dimension: substrate and metabolites resonate all at slightly different frequencies, making the spectrum of each molecule its peculiar fingerprint. In this context, ^13^C is the most adapted nucleus because of its ubiquitous presence in the vast majority of biomolecules, and its large chemical shift dispersion that allows to easily differentiate the various species. Nevertheless, ^13^C MRSI pays the heavy toll of low sensitivity and poor temporal/spatial resolution^4^. Indeed, the low natural abundance (i.e. 1% of carbon exist as ^13^C), the low physiological concentration of the biomolecules (1000-fold lower relative to water) and the 4-time lower magnetic energy of a ^13^C nucleus with respect to a ^1^H one when immersed in a magnetic field, impose severe limitations when ^13^C MRSI is performed at thermal equilibrium and without exogenous labeling^5^.

^13^C hyperpolarization methods^6,7^ generate ex-situ isotopically labeled and injectable compounds with an MR signal enhancement of 10^4^ – 10^5^ orders of magnitude (i.e., hyperpolarized molecular contrast agents). As a result, hyperpolarized (HP) ^13^C MRSI can be collected with unprecedented dynamic and spatial resolution^8,9^, giving access to metabolic fluxes in real-time and in vivo.

Over the past two decades, this methodology has gained great significance in preclinical studies thanks to three hyperpolarization techniques: PHIP-SAH (ParaHydrogen-Induced Polarization-Side Arm Hydrogenation)^10,11^, SABRE (Signal Amplification by Reversible Exchange)^12^, and dDNP (dissolution Dynamic Nuclear Polarization)^13^. So far, thanks to its molecular versatility^14^, straightforward biocompatibility (no employment of metal complex catalysts and toxic solvents), and superior polarization level at time of injection^15–17^, only dDNP-produced HP molecular contrast agents (MCAs) have made it into humans. In particular, dDNP hyperpolarized [1-^13^C]pyruvate has been used to diagnose cancer and detect treatment response^18–20^, and hyperpolarized [^13^C, ^15^N]urea has been used for tissue perfusion purposes^21,22^.

Nevertheless, this methodology struggles to enter everyday clinical practice. One of the reasons why broad consensus among clinicians is still missing lies in the technical complexity that characterizes hyperpolarization via dDNP^23^. Indeed, the process takes place in the solid-state at cryogenic temperature (0.8–1.4 K) and high magnetic field (3.35–7 T). The nuclear signal enhancement is the consequence of off-resonance microwave excitation of specific Electron Polarizing Agents (EPAs) dissolved, together with the MCA, in a glassing solvent, to form an amorphous solid upon freezing. These experimental conditions of magnetic field, low temperature, and microwave irradiation are provided by costly and technically demanding hardware known as “dDNP polarizer”. The solid-state polarization step is then followed by the dissolution one: a sudden melting and dilution of the sample by means of super-heated buffer. A main drawback is that, after dissolution, all HP ^13^C MCAs are short-lived (i.e., minute-long half-life of the HP state).

Although the half-life of HP ^13^C MCAs is orders of magnitude longer when kept at cryogenic temperatures^24,25^, samples prepared with traditional EPAs, such as trityl, cannot be extracted as a frozen solid from the dDNP polarizer without losing their HP state. Indeed, paramagnetic relaxation at low magnetic field (i.e., when moving the sample far from the magnet’s isocenter) would destroy the high nuclear spin order within few milliseconds^26^.

Currently, should one be willing to equip an MRI facility with hyperpolarization, the only way is to place a dDNP polarizer on site. Lifting these restrictions and making ^13^C hyperpolarization transportable, as already shown for ^129^Xe on the scale of several hours^27^, would allow centralized production of HP substrates followed by transport to multiple locations for HP MRI use, in a similar fashion as for ^18^F-fluorodeoxyglucose (^18^F-FDG) PET tracers^28^.

So far, to circumvent this shortcoming, research has pursued three different approaches. The first is to use UV-induced labile radicals that can be quenched, after the DNP process has happened in the solid state inside the polarizer, by heating up the sample above 190–200 K by means of a thermalization process^29–32^. The second is to physically separate carbon nuclei from the EPAs by utilizing differences in solubility of radicals and ^13^C-MCAs^33^. The third is to physically separate carbon nuclei from EPAs by grafting the radicals inside purpose synthesized porous polymers that can absorb a solution containing the substrate of interest^34^.

HP substrates polarized with UV-induced labile radicals, as well as the porous polymers, can have the advantage of being readily biocompatible. Indeed, the most used radical precursors are endogenous alpha-keto acids. Moreover, differently from synthetic radicals, the natural radical scavenging upon temperature increase of the sample removes the need for filtration prior to injection, when a radical free solution is required^8^.

As of today, to the best of our knowledge, all attempts of transport have been limited to a very small scale and to the level of proof-of-principle in vitro experiments^30,31,33^. In this paper, combining UV-induced labile radicals, a robust strategy for sample polarization, thermalization, extraction and storage^35^, we demonstrate the successful across-cities in vivo HP ^13^C-MRI pre-clinical experiments for a perfusion/angiographic ([1-^13^C]HP001, i.e. bis-1,1-(hydroxymethyl)-[1-^13^C]cyclopropane-d_8_) and a metabolic ([U-^13^C, d_7_]glucose) HP MR contrast agent.

## Results

### ESR and DNP characterization

**Fig. 1:**
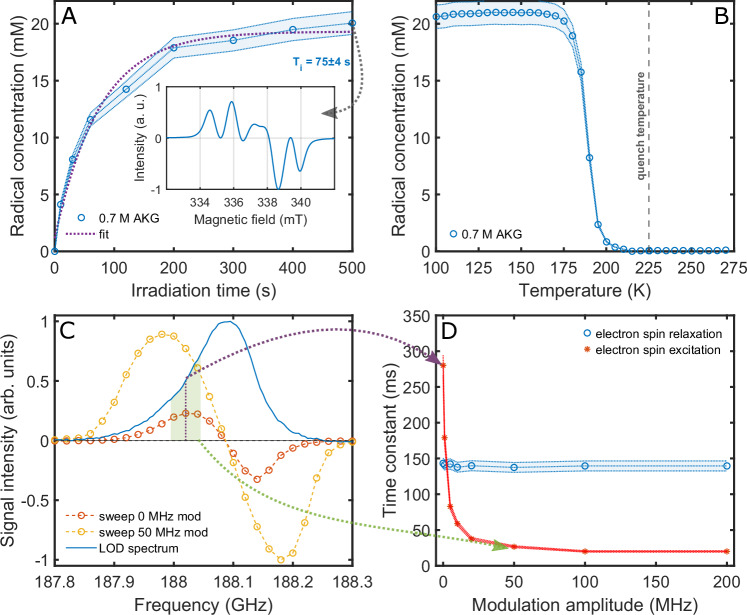
Electron spin resonance (ESR) and dynamic nuclear polarization (DNP) properties of the reference sample of the study. Typical (*n* = 1) radical generation curve measured by means of X-band ESR spectroscopy at 77 K in a small quartz dewar for a glucose sample containing alpha-ketoglutairc acid (AKG) as radical precursor; 15 × 10.0 ± 0.5 µL frozen beads of sample solution were irradiated in liquid nitrogen with high power (35 W/cm^2^) UV-light for increasing time intervals, and measured via ESR at the end of each interval; the error band (light blue area) is ±5% of the measured radical concentration of each data point and comes from spectrometer calibration; a mono-exponential curve (violet dotted line) was fitted to the data, its mathematical expression is $C\left(t\right)={C}_{\infty }\left(1-\exp \left(-t/{T}_{i}\right)\right)$, where $t$, ${T}_{i}$, ${C}_{\infty }$, $C\left(t\right)$ represent the time of irradiation, the irradiation time constant, the concentration at saturation, and the concentration at time ${{\rm{t}}}$, respectively. ${T}_{i}$ was 75 ± 4 s with *R*^*2*^ = 0.95; the inset shows the 1st derivative of the ESR spectrum corresponding to the last data point (*n* = 1) of the time-course (**A**). Typical (*n* = 1) radical’s quench temperature measured by means of X-band ESR spectroscopy; the ESR spectrometer was equipped with a Variable Temperature Insert (VTI) and one bead was transferred inside the sensitive area of the VTI after complete UV-irradiation; the temperature was increased in steps of 5 K and the ESR signal measured after thermal stabilization of the system; the gray dashed line indicates when no more signal could be measured from the sample; the error band (light blue area) is ±5% of the measured radical concentration of each data point and comes from spectrometer calibration; (**B**). Typical (*n* = 1) sample’s DNP and ESR spectra at 6.7 T and 1.2 K; after loading the sample inside the dDNP polarizer, its ESR spectrum at high magnetic field was measured by means of the Longitudinal Detected (LOD)-ESR technique (light blue line); the ^13^C signal enhancement as a function of the microwave irradiation frequency (i.e. the DNP spectrum) was also measured with (yellow circles) and without (orange circles) microwave frequency modulation; the positive DNP maximum (violet dotted line) and the portion of irradiated ESR spectrum corresponding to 50 MHz of frequency amplitude modulation (green rea) are also indicated (C). To investigate the effect of microwave frequency modulation (FM) on the radicals’ electron spins, their dynamic upon excitation (microwaves go ON, red circles) and relaxation (microwaves go OFF, blue circles) was also measured for increasing values of FM; the error bands (light blue area for spin relaxation measurements and light red area for spin excitation measurements) come from the accuracy of the fit for each measurements (see “Methods” and Supplementary Information) (**D**). The violet and green arrows connecting panel **C** and panel **D** represent microwave irradiation conditions at which the two specific data points in panel D were acquired, i.e. irradiation at 188.02 GHz with FM at 0 MHz and 50 MHz, respectively. Source data are provided as a Source Data file.

A full characterization of the samples was first carried out by means of Electron Spin Resonance (ESR) and solid-state DNP measurements. As an example, in Fig. 1, we summarize the results of the reference sample of this study: a solution of 2 M [U-^13^C, d_7_]glucose (from now on referred to as glucose) and 0.7 M alpha-ketoglutaric acid (from now on referred to as AKG) dissolved in D_2_O:d_8_-glycerol 1:1 (v/v) (from now on referred to as deuterated solvent). In Fig. 1A, we used X-band ESR spectroscopy to quantify the radical generation upon UV-light irradiation in liquid nitrogen^36^ of 15 × 10 µL frozen beads of sample solution (see “Methods” for details). A UV-irradiation at 35 W/cm^2^ was sufficient to saturate the radical generation around 20 mM after 300 s, with a quantum yield of almost 3%. The inset shows the derivative of the ESR spectrum at the end of the irradiation. The latter was mainly characterized by the dipolar coupling of the unpaired electron localized on the secondary carbon atom with the adjacent 2H group^29^. As earlier demonstrated^36^, we fitted the radical generation growth with a mono-exponential curve $C\left(t\right)={C}_{\infty }\left(1-\exp \left(-t/{T}_{i}\right)\right)$, where $t$, ${T}_{i}$, ${C}_{\infty }$, $C\left(t\right)$ represent the elapsed time of irradiation, the irradiation time constant, the concentration at saturation, and the concentration at time $t$, respectively. ${T}_{i}$ was 75 ± 4 s with R^2^ = 0.95. Although the ${T}_{i}$ estimation was accurate enough to evaluate the minimum irradiation time needed to saturate the radical concentration in the sample (i.e., <300 s), the quality of the fit was most likely affected by the fact that all beads were irradiated at once. R^2^ was higher when irradiating one single bead at a time^36^.

In Fig. 1B, the radical quench temperature was measured using the X-band ESR spectrometer equipped with a Variable Temperature Insert (VTI). The result was a steep drop in the radical concentration between 175 K and 200 K. No ESR signal could be detected above 225 K (Fig. S1).

Figure 1C shows the ESR and DNP properties of the sample at 1.2 K and 6.7 T. To perform this characterization, a home-built dDNP polarizer was used. The latter included a cold bore superconductive magnet equipped with a cryogenic solid-state NMR probe able to detect the ^13^C signal as well as to shine microwaves onto the frozen sample by means of a waveguide and a non-resonant cavity (see “Methods” for details about the dDNP polarizer). From now on, the cryogenic NMR probe with microwave irradiation capability will be referred to as the dDNP probe^31,37^. The high field ESR spectrum was measured by means of a LOngitudinal Detection ESR (LOD-ESR) probe slit inside the dDNP one^36,38^, and it was overlayed to the ^13^C NMR signal as a function of the microwave irradiation frequency, i.e., the DNP spectrum. It is important to notice that a LOD-ESR setup is easily implementable on a dDNP polarizer with no major hardware modifications. Indeed, the LOD-ESR technique measures the electron spin signal along the same axis of the static field B_0_ using a solenoidal pickup coil and with no need for a resonant cavity (see “Methods” for details). For further details, see the seminal work by Granwehr et al.^39^. The DNP spectrum was measured twice by keeping all experimental parameters unchanged except for the microwave frequency output, kept constant in the first case (mono-chromatic irradiation), and swept at a rate of 1 kHz around the central frequency by ±25 MHz (50 MHz of microwave frequency modulation) in the second. Both DNP spectra nicely overlapped with the ESR one, showing that the enhancement mechanism happened only for microwave frequencies where electron spins were also resonating, a clear sign of DNP by Thermal Mixing or Cross-Effect^40,41^. Interestingly, the DNP signal increased by 4-fold when microwave frequency modulation (FM) was used. To further investigate this observation, we measured, by means of LOD-ESR, the electron spin dynamic of the EPA at the non-modulated DNP positive maximum (i.e. 188.02 GHz) for increasing FM values (Fig. 1D).

While the characteristic time constant of the electron spins system upon turning OFF the microwaves (i.e., electron spins relaxation, T_eR_) stayed constant around 150 ms, the dynamic upon turning ON the microwaves (i.e., electron spins excitation T_eE_) decreased from 280 ± 15 ms, for monochromatic irradiation, to 25 ± 2 ms, for 50 MHz of FM. No significant decrease was observed for higher values of FM. It is worth noting that we prefer to refer to relaxation and excitation instead of the commonly used “electron T_1_” and “saturation constant” because the dynamic we observed is affected by spectral diffusion.

### Polarization at optimal conditions and thermalization

**Fig. 2:**
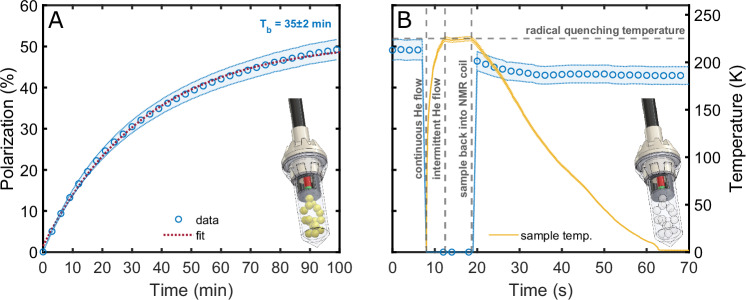
Reference sample polarization and thermalization. Typical (*n* = 1) ^13^C signal time-course (blue circles) at best microwave irradiation conditions (frequency = 188.18 GHz, frequency amplitude modulation = 50 MHz), 6.7 T and 1.2 K; a mono-exponential curve (violet dotted line) was fitted to the data, its mathematical expression is *P*(*t*) = *P*_∞_(1 − *exp*(−*t*/*T*_*b*_), where *t*, T_b_, P_∞_, ${{\rm{P}}}\left({{\rm{t}}}\right)$ represent the polarization time, the polarization time constant, the polarization at infinite time, and the polarization at time $t$, respectively. ${{{\rm{T}}}}_{{{\rm{b}}}}$ was 35 ± 2 min with *R*^*2*^ = 0.97 (**A**). Typical (*n* = 1) ^13^C signal time-course (blue circles) during sample thermalization with overlayed temperature profile (yellow line); the vertical dashed gray lines indicate the different steps of the thermalization process; the horizontal dashed gray line indicates the set temperature threshold during heating by He gas blowing (**B**). In both panels, the error band (light blue area) is ±5% of the measured polarization value of each data point and comes from NMR spectrometer calibration. The two insets show the sample appearance at the bottom of the custom fluid path (CFP) before (colored frozen beads) and after (transparent frozen beads) the thermalization process; the color loss was due to the recombination of radicals into diamagnetic species. Source data are provided as a Source Data file.

Afterwards, the ability to hyperpolarize the sample and make its nuclear spin order long-lived with minimal losses was investigated (Supplementary Video 1). Figure 2 shows the sample polarization buildup (6.7 T, 1.2 K) and thermalization above the radical quenching temperature. A Custom Fluid Path (CFP)^31,38^, equipped with a temperature sensor, was used to seal and load the sample inside the dDNP polarizer, and monitor its heating profile during thermalization. The reference sample was first polarized at best microwave conditions (carrier frequency 188.18 GHz, output power 50 mW, FM 50 MHz) obtaining a solid state ^13^C polarization of 48* ±* 2 % after 90 min (panel A). Data points were fitted with a mono-exponential curve $P\left(t\right)={P}_{\infty }\left(1-\exp \left(-t/{T}_{b}\right)\right)$, where $t$, ${T}_{b}$, ${P}_{\infty }$, $P\left(t\right)$ represent the time of polarization, the polarization time constant, the polarization at infinite time, and the polarization at time $t$, respectively. ${T}_{b}$ was 35* ±* 2 min with R^2^* =* 0.97. The left inset displays a sketch of the bottom part of the CFP (i.e., what is immersed in liquid He and sits inside the NMR coil) during polarization. The sample beads are colored to indicate the presence of radical.

After polarization, the sample was thermalized using room temperature He gas (panel B). This procedure allowed us to get rid of more than 99 % of the initial radical concentration (Fig. S2) in a reproducible and robust manner, by limiting polarization losses to no more than 20% of the maximum value achieved during DNP. The right inset displays the sample as transparent beads because the thermalization procedure and consequent radical annihilation made them lose their characteristic color.

### Extraction from dDNP polarizer and storage

**Fig. 3:**
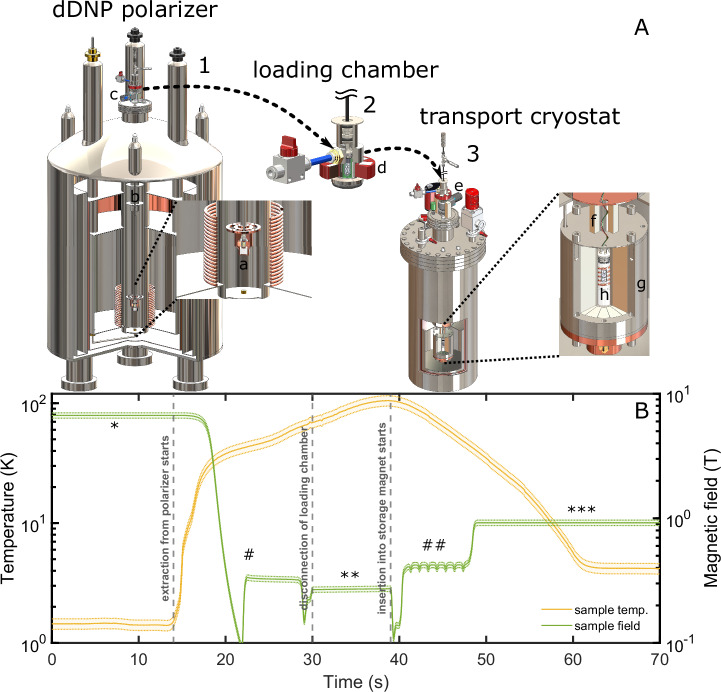
Radical free HP reference sample extraction and storage procedure. Sketch of sample extraction and storage step-by-step procedure: step 1—after thermalization, the sample is manually lifted from the microwave cavity (a), along the dDNP probe (b), up to a removable loading chamber (d); step 2—the dDNP polarizer’s gate valve (c) is closed and the loading chamber disconnected from the polarizer and docked to the transport cryostat; step 3—the gate valve of the transport cryostat (e) is opened and the he sample pushed through a magnetic rail (f) down to a liquid He cooled permanent magnet (g), to reach a solenoidal NMR coil (h) (**A**). Magnetic field (green line) and temperature (yellow line) experienced by the sample during these operations; errors on the measurements are reported as shaded areas around the data points; the vertical dashed gray lines indicate the onset of each of the three main steps; special characters indicate regions of constant magnetic field: (*) polarizer superconductive coil; (#) Halbach array along the polarizer probe; (**) Halbach array around loading chamber; (##) Halbach array along the portable cryostat’s probe; (***) 1 T NMR quality permanent magnet. The error bands, light yellow for the temperature and light green for the magnetic field, are ±10% and ±5% of the measured value and come from the respective measurement instrument accuracy (see “Methods”). Source data are provided as a Source Data file (*n* = 1) (**B**).

The extraction from the dDNP polarizer (Fig. 3A) into a purpose-built transportable liquid He cryostat with NMR capability^35^ took approximately 50 s and happened in three steps (Supplementary Video 2). Firstly, the sample was raised from the isocenter of the superconductive magnet to the sample loading chamber. Secondly, the dDNP polarizer gate valve was closed, the loading chamber with sample inside removed and docked on top of the portable cryostat. Thirdly, the gate valve of the portable cryostat was opened and the sample quickly lowered into the liquid He cold storage permanent magnet. The temperature and magnetic field experienced by the sample during this procedure are reported in Fig. 3B. It is important to notice that different arrangements of permanent magnets constituting a “magnetic rail” were mounted around the dDNP probe and inside the portable cryostat to always keep the sample in a magnetic field of at least 100 mT, all along the extraction path from the isocenter of the 6.7 T superconductive magnet to the one of the 1 T permanent storage magnet. Construction details of the permanent magnets arrangements were described earlier^31,35^.

During this process, the sample’s temperature quickly increased from 1.2 K at the beginning of the extraction to approximately 30 K when the vial was raised into the loading chamber; displacing the loading chamber from the polarizer and docking it to the top of the portable cryostat took approximately 20 s and generated a further temperature increase to 100 K, but at a lower rate; once inside the portable cryostat, it took further 20 s to cool the sample down to 4.2 K.

It is worth noting that transferring the HP sample from the transportable cryostat back inside the DNP polarizer showed that, within 5% error, the signal intensity was equal to the one from the last acquisition before extraction, proving the procedure to be essentially loss-free.

**Fig. 4:**
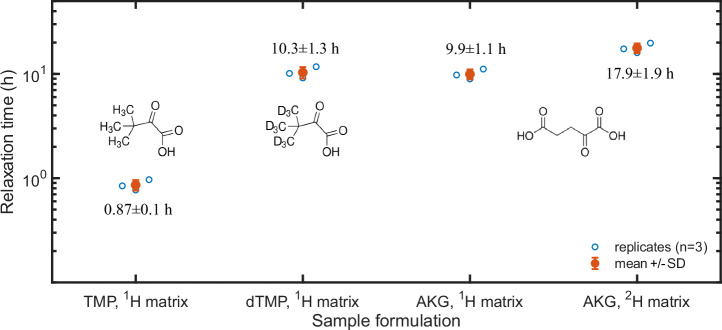
^13^C sample relaxation at storage conditions. ^*1*3^C nuclei solid-state relaxation time constant at 1 T and 4.2 K for samples containing 2 M [U-^13^C, d_7_]glucose that have been polarized, thermalized, extracted, and stored inside the portable cryostat as a function of the molecular structure of the radical precursor and the deuteration level of the glassy solvent matrix. Four formulations are reported: 0.7 M of trimethylpyruvic acid dissolved in H_2_O:glycerol 1:1 (v/v) (TMP, ^1^H matrix); 0.7 M of d_9_-trimethylpyruvic acid dissolved in water:glycerol 1:1 (v/v) (dTMP, ^1^H matrix); 0.7 M of alpha-ketoglutaric acid dissolved in H_2_O:glycerol 1:1 (v/v) (AKG, ^1^H matrix); 0.7 M of alpha-ketoglutaric acid dissolved in D_2_O:d_8_-glycerol 1:1 (v/v) (AKG, ^2^H matrix). Each tick on the x-axis represents a sample formulation. For each formulation 3 different samples (*n* = 3) were measured. For each formulation, replicates are reported as blue circles, the mean value ± standard deviation is reported as a red dot with its error bar. Source data are provided as a Source Data file.

Polarization, extraction and storage were repeated on different glucose sample preparations in terms of radical precursor molecule and level of deuteration of the solvent (i.e. glycerol:water 1:1 (v/v)) (Fig. 4). While minimal differences were measured in radical yield, quench temperature, and DNP performance^29^ (for details see Figs. S3 and S4), these 2 parameters had a huge impact on the relaxation time at storage conditions. The residual radical concentration after thermalization was approximately 0.5% of the initial value regardless of radical precursor. The glucose ^13^C *T*_*1*_ of the reference sample at 4.2 K and 1 T measured 0.87 ± 0.01 h for trimethylpyruvic acid (TMP) as radical precursor and in a protonated solvent, 10.3 ± 1.3 h for d_4_-trimethylpyruvic acid (dTMP) as radical precursor and in a protonated solvent, 9.9 ± 1.1 h for AKG as radical precursor and in a protonated solvent, and 17.7 ± 1.9 h for AKG as radical precursor and in a deuterated solvent. Figure S5 shows the decay of each sample during the liquid He holding time of the transportable cryostat (7 h).

### Hyperpolarized 13C imaging

The sample formulation providing the longest relaxation time (2 M glucose and 0.7 M AKG dissolved in d_8_-glycerol:D_2_O) was used to perform HP MRSI in vivo. The experiment took place at the MR Research Center of Aarhus University Hospital (Aarhus, Denmark), where we transported a long-lived HP sample from the Technical University of Denmark (Kgs, Lyngby, Denmark). The transport took 5 h and covered 320 km (Supplementary Video 3). Once on site, we performed the dissolution from the transportable cryostat and injected a rat through its tail vein, previously prepared in a 3 T scanner, with 1 mL of HP glucose solution at 60 mM (Supplementary Video 4).

MRSI was acquired with the observation frequency centered on the c2-c5 glucose multiplet. Considering losses during transport, an elapsed time of 30 s between dissolution and beginning of the MR acquisition, and a glucose liquid-state T_1_ of 15 s^31^, we estimated a ^13^C polarization between 5 and 7 % at time of injection. As a first attempt and because of the broad chemical shift of uniformly ^13^C,d_7_ labeled glucose, the entire HP signal was used to acquire one single image (Fig. S7).

**Fig. 5:**
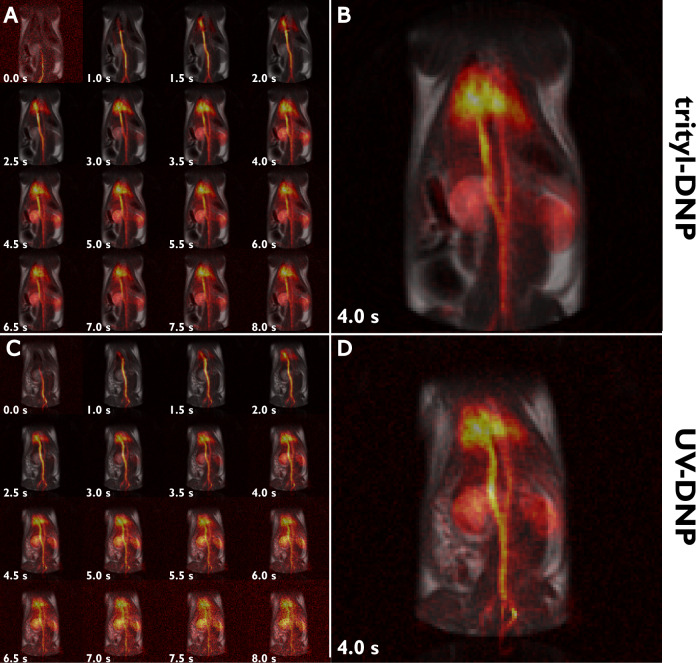
Rat angiography using hyperpolarized HP001(bis-1,1-(hydroxymethyl)-[1-13 C]cyclopropane-d8). The first rat (*n* = 1) was injected with the substrate polarized on-site with a polarizer next to the MRI scanner, at Aarhus University Hospital, and using stable trityl radicals; polarization at injection was approx. 54%. In the time series one image was acquired every 0.5 s (**A**). A zoom of frame #8, corresponding to the max coverage of the cardiovascular system before the signal starts fading out, is also reported (**B**). The second rat (n = 1) was injected with the substrate polarized at DTU, Lyngby thermalized to make it radical free and long relaxing, and transported over 320 km for 5 h to Aarhus University Hospital; polarization at injection was approx. 24%. In the time series one image was acquired every 0.5 s (**C**). A zoom of frame #8, corresponding to the max coverage of the cardiovascular system before the signal starts fading out, is also reported (**D**). The acquisition time for each frame with respect to the beginning of the acquisition is also reported.

The experiment was repeated hyperpolarizing a compound with longer liquid-state *T*_*1*_ and an isolated singlet ideal for HP imaging: HP001(bis-1,1-(hydroxymethyl)-[1-^13^C]cyclopropane-d_8_). The DNP sample was prepared in the same way, except that the 2 M glucose was replaced by 5 M of HP001. Two experiments were performed the same day on two healthy rats. The first rat was imaged by hyperpolarizing HP001 on site and using trityl radicals and a SpinAligner polarizer^16,42^. The liquid-state signal of a 150 mM single peak ^13^C solution was used to acquire a complete angiogram by means of a gradient echo sequence with frames every 0.5 s. Figure 5A shows the rat cardiovascular system’s highlight starting from the tail vein injection. Complete coverage of heart and kidneys was achieved at frame #8 (Fig. 5B), after 4 s from injection. Afterwards, the signal slowly started to fade out.

The hyperpolarized angiogram was repeated on a second rat using the hyperpolarized signal from the transported sample (Fig. 5C). Complete coverage of heart and kidneys was achieved at frame #8 as well (Fig. 5D). For both experiments, 1 mL of HP solution was used for the tail vein injection. A second syringe was brought to a benchtop NMR spectrometer to estimate polarization at injection. With a *T*_*1*_ of 82 ± 2 s (measured at 1 T on the benchtop spectrometer) and a transfer time of 30 s the ^13^C polarization at injection was estimated to be 52% and 24% for the polarized-on-site and transported sample, respectively.

## Discussion

Our results clearly show that we pushed transportable hyperpolarization robustly to proof-of-concept, paving the way for democratizing access to state-of-the-art ^13^C-HP MRSI by enabling centralized production and distribution similar to what is done with [^18^F]FDG-PET. A thorough handling of all key parameters (i.e., high enough radical concentration, efficient ^13^C DNP above 50% polarization, minimal losses during thermalization, extraction and storage) allowed us to successfully perform HP MRSI in vivo with a contrast agent polarized off-site. Although the angiogram acquired using the transported MCA showed lower SNR, the image contrast was high enough to visualize the same main anatomical elements of the rodent. Half of the polarization was lost during the full experimental procedure and 5 h transport. Indeed, it was earlier demonstrated that, hyperpolarizing HP001 using UV-radicals at 6.7 T/1.2 K and dissolving it on-site generated close to 50% ^13^C polarization in the liquid state^29^.

If HP transportation aims at covering a shorter distance, liquid nitrogen could be used. At 77 K and 1 T, a carbon T_1_ of more than 30 min was measured on the reference sample (Fig. S11), with the advantage that, at these experimental conditions, the transportable cryostat can be half the size, the weight, and the cost.

A very important outcome of this study is shown in Fig. 4. Here, differently from what was explained in the introduction, we clearly show that to make the high spin order of a sample polarized via DNP long-lived for hours, moderate magnetic field, low temperature and radicals’ absence/negligible leftover are necessary but not sufficient conditions. The sample’s solid matrix composition also plays an important role. Methyl group driven relaxation via tunneling^43^ strongly influenced ^13^C nuclei T_1_ at 1 T and 4.2 K. When radical precursor molecules with methyl group were employed (i.e. TMP) the glucose relaxation time did not exceed 1 h. Eliminating methyl group driven relaxation by replacement of the precursor molecule with a methyl group free one (i.e. AKG), or mitigating it via deuteration of the radical precursor molecule (i.e. dTMP), extended the MCA relaxation time by 1 order of magnitude. Indeed, deuteration significantly reduces the rate of quantum mechanical tunneling in methyl groups because the heavier deuterium atoms make tunneling through a potential barrier much less probable^44^. Replacing the water:glycerol solvent with its deuterated counterpart further improved the T_1_ value by a factor of two. Carbon polarization can leak through the surrounding nuclei via spin diffusion, which is still active even in absence of radical-driven paramagnetic relaxation^45,46^. After thermalization, the abundant nuclear species in our samples (i.e. ^1^H and ^2^H) are completely depolarized because of fast spin diffusion during heating and can act as a polarization sink for ^13^C nuclei. Nevertheless, since γ(^2^H)* <* γ(^1^H), spin diffusion-assisted leakage of ^13^C polarization is less efficient for deuterated glassing matrices^47^.

The presence of ^1^H and ^2^H nuclei at thermal equilibrium in the sample had another important consequence: it made the presence of permanent magnets along the extraction path a key requirement for a successful experiment. Exposing the thermalized samples to a magnetic field of at least 100 mT, allowed us to avoid spectral overlap of the ^13^C NMR line with the ^1^H or ^2^H ones. A spectral overlap would lead to energy exchange between the different nuclear pools draining polarization from carbon nuclei by means of the mechanism known as low-field Thermal Mixing^24^.

Because of dTMP limited availability and unknown biocompatibility, our radical precursor of choice for the in vivo experiments became AKG, that was introduced by Gaunt et al.^48^. Nevertheless, at same UV-irradiation conditions, we measured a quantum yield of only 3% instead of the 30% earlier reported^48^. The latter was due to the absence of lactic acid in our sample preparation that can act as electron acceptor during the photo-excitation process^49^. At the same time, working with a UV-radical showing a more complex spectrum with respect to the single line of TMP and dTMP, led us to interesting observations during the annihilation process. Figure S1 shows that the UV-irradiated AKG spectrum changed appearance around 175 K, when the quenching process started: first it became narrower and then lost one 1/2 nuclear spin coupling evolving from a quadruplet to a triplet. Around 190 K, the radical concentration dropped by half, and at 200 K the radical spectrum showed a single peak to then disappear above 225 K. This evolution of the signal suggested that the radical quench process either happens in more steps, or more than one radical species are generated in the first place. It is important to notice that the signal evolution was not reversible: cooling back down the sample did not lead to the recovery of any feature in the spectral appearance. We are currently running more experiments to quantify this phenomenon.

Although DNP performances were similar to what we obtained earlier using TMP and dTMP^29^, FM played an even more crucial role to achieve high polarization in the solid state using AKG. The reason for that was the surprisingly slow electron spin dynamics upon microwave excitation (Fig. 1D). Without FM, excitation was two times slower than relaxation. The consequence was poor spectral diffusion leading to only a small portion of the radical ESR spectrum to participate to the DNP mechanism^50,51^. Since the characteristic time constant ${\tau }_{D}$ for the electron spins excitation to spread across the entire ESR line depends on the radical spectral width $D$ and the diffusion coefficient $\triangle \left(\omega \right)$ as ${\tau }_{D}={D}^{2}/4\triangle \left(\omega \right)$^51^, FM artificially decreased $D$, making spectral diffusion faster with respect to ${T}_{{eR}}$ that remained constant instead (see Fig. S9 for details). DNP improved until the excitation time constant reached a plateau for 50 MHz of FM. Higher values of FM led to a slight performance decrease of the DNP because electron spins contributing the enhancement with opposite polarity started to be involved.

A more quantitative analysis of this phenomenon is beyond the scope of this paper and will be the object of future work. Nevertheless, as a comparison, Figure S10 shows the LOD-ESR and DNP investigation of the properties of a sample that does not benefit from FM: 2 M glucose dissolved in glycerol:water 1:1 (v/v) doped with 20 mM of AH111501 trityl radical. The main difference with respect to the AKG-glucose sample was the 5-time faster excitation dynamics with respect to relaxation, already for monochromatic microwaves irradiation.

It is dutiful to discuss the limitations of this technique, thus to mention the case of [1-^13^C]pyruvic acid. Despite high initial polarization^36^, robustness and ease of operation of our CFP based methodology, a thermalization step > 10 s always depleted at least 80% of the hyperpolarized [1-^13^C]pyruvic acid signal. Moreover, the remaining spin order did not survive manual extraction. These two observations were the consequence of fast relaxation arising from the presence of methyl groups in the vicinity of ^13^C. Using [d_4_, 1-^13^C]pyruvic acid instead, and mixing it in equal volume to deuterate glycerol:water mitigated the problem. Polarization at extraction and losses like the ones for the glucose sample were measured. Nevertheless, even at 4.2 K, the carbon T_1_ at storage conditions did not exceed 90 min (Fig. S12). Therefore, HP MR contrast agents with methyl groups in their molecular structure might benefit from a milliseconds-long thermalization and extraction procedures. Moreover, it was earlier demonstrated that changing the pyruvic acid’s glass properties by means of annealing^24^, can prolong its carbon half-life by one order of magnitude.

In conclusion, we demonstrate that under certain circumstances hyperpolarization via DNP can be transported, and the high spin order preserved well enough to conduct in vivo experiments with a contrast very similar to what can be obtained by means of an on-site polarizer. We were successful in performing in vivo ^13^C HP MRI perfusion with good SNR using an HP001 5 h after its generation off-site. Differently, we did not yet succeed in repeating the same experiment for metabolic imaging of [1-^13^C]pyruvic acid. Deuteration of the molecule allowed us to obtain after extraction and storage a polarization and relaxation time around 40% and 90 min, respectively. The latter would allow shorter (i.e. within the same city) transport, and it is currently object of investigation at the University of Eastern Finland by Dr Mikko Kettunen.

## Methods

### Sample preparation, radical generation and quench temperature

All chemicals were purchased from Sigma-Aldrich (Brøndby, Denmark) excepted for the radical precursor deuterated trimethylpyruvic acid (dTMP) that was synthesized in house^29^, and the AH111501 trityl radical purchased from Polarize ApS (Frederiksberg, Denmark). The substrate (2 M of [U-^13^C, d_7_]-D-glucose or 5 M of HP001(bis-1,1-(hydroxymethyl)-[1-^13^C]cyclopropane-d_8_)) and the radical precursor (0.7 M of TMP or 0.7 M of dTMP or 0.7 M of AKG) were dissolved in the solvent solution (glycerol:water 1:1 (v/v) or d_8_-glycerol:D_2_O 1:1 (v/v)) to obtain a final sample volume of 150  ±  5 µL; the latter was sonicated at 40 °C for 5 min to efficiently degas the sample and improve the glass quality after freezing. 15 × 10.0  ±  0.5 µL droplets were poured in liquid nitrogen to form frozen beads. The frozen sample was transferred to a quartz Dewar (Magnettech, Berlin, Germany) filled with liquid nitrogen for UV irradiation. The irradiation set up was extensively described earlier^36^. UV-light was shined in steps on the sample up to 500 s and using two broad-band source (Dymax BlueWave 75, Connecticut, USA) at full power (i.e., 35 W/cm2). Radical concentration was measured immediately after each irradiation step by inserting the tail of the quartz Dewar in the cavity of an X-band spectrometer (Miniscope MS 5000, Magnettech, Berlin, Germany). The spectrometer parameters, kept constant for all measurements, were optimized to avoid any saturation or line broadening of the ESR signal, i.e. center of the sweep = 338 mT; sweep range = 20 mT; sweep time = 20 s; modulation frequency = 100 kHz; modulation amplitude = 0.1 mT; microwave power = 0.2 mW^36^. The software used for the acquisition was ESR Studio from Magnettech.

To quantify the radical quench temperature of the sample, the X-band ESR spectrometer was equipped with a Variable Temperature Insert (VTI) and 1 UV-irradiated sample bead transferred into it. The radical concentration profile as a function of temperature was characterized from 100  to 275 K in steps of 5 K. To consider the Boltzmann factor and relate the data points’ value to the number of spins only, the signal intensity was multiplied by the temperature at which it was acquired.

The glucose and HP001 samples prepared in glycerol:water with 20 mM AH111501 trityl radicals were not UV-irradiated.

### Custom fluid path (CFP) design

The CFP was built using two concentric tubes (an outer lumen—black—and an inner lumen - red - ending with a nozzle - green-, as shown in Fig. 2) that allow gas or liquid to flow from a dispenser onto the sample and out of the sealed environment (all design details and operation’s principles were reported earlier^31^). A pierced half cylinder (black cut view in the figure) was used to avoid obstruction of the nozzle’s output from direct contact with the sample that sits in the vial (transparent component in the figure). For this study, the CFP was equipped with a Cernox temperature sensor (CX-1030, Lake Shore Cryotronics, Westerville, USA) to monitor sample’s temperature during thermalization and extraction.

### Solid-state DNP and LOD-ESR

All DNP and LOD-ESR measurements were performed on a homebuilt dDNP polarizer (6.7 T magnet from Magnex, Oxford, UK) working at 1.15 ± 0.05 K conceptually similar to the idea introduced in 2003^13^. The original system (3.35 T) was retrofitted to work at higher field and employ the CFP technology^36,37^. Indeed, the polarizer was equipped with a 94 GHz solid-state source (Polarize Aps, Copenhagen, Denmark) coupled to a 200×2R4 frequency doubler (VDI, Charlottesville, VA, USA), which provided an output power of 100 mW at 188 GHz. The source, digitally controlled through NI-DAQ device USB-6525 (National Instruments, Austin, TX, USA) had a tuning range of ±1 GHz and the possibility to modulate the output frequency at a rate up to 10 kHz and with an amplitude of up to 500 MHz. All ^13^C NMR acquisitions were performed using a Varian INOVA console (Palo Alto, CA, USA) connected to a low temperature dDNP probe remotely tuned to 71.8 MHz and equipped with permanent magnets along the central loading tube^31^. The dDNP probe was placed inside the Variable Temperature Insert (VTI) of the polarizer.

For LOD-ESR measurements a home-built spectrometer was used. The spectrometer was controlled using a purpose made software in LabView 2019 (National Instruments)^36,38^. For radical spins relaxation and saturation time constant measurements the output power of the microwave source was modulated at 0.5 Hz between 0 mW and 50 mW. The rate was low enough to record the full-time evolution of the electron spins during saturation and relaxation; the signal was averaged 256 times. Extraction of the relaxation and excitation time constants (*T*_*c*_) were performed by fitting the signal evolution in the time domain to the equation $S\left(t\right)=A\left(\exp \left(-t/{T}_{c}\right)-\exp \left(-t/\tau \right)\right)$ where *A* is a proportionality factor depending on the sample properties and measuring parameters and $\tau$ the time constant of the measuring split solenoid coil. By measuring the time constant $\tau$ exciting the coil through a step function, *A* and *T*_*c*_ were the only free parameter of the fit. For radical spectrum recording, the microwave frequency was increased from 187.8 GHz to 188.3 GHz in steps of 1.25 MHz and the output power modulated at 4.8 Hz between 0 mW and 50 mW, and this signal fed into a lock-in amplifier. For each frequency step the demodulated signal was integrated for 20 s in the time domain, equivalent to set the low pass filter of the lock-in to 0.05 Hz.

### Thermalization, extraction and NMR measurements from the transportable cryostat

To thermalize the sample and quench the radicals, the CFP was raised by 10 cm pulling the sample out of the NMR detection coil and positioned above the liquid He level. At this point, microwaves were switched off, the top part of the CFP was connected to a room temperature He gas dispenser, equipped with a pneumatic valve, and the flow was controlled by a feedback loop on the sample temperature. He gas was blown continuously for approximately 4 s at 10 bar until the radicals’ quench temperature of 225 K was achieved and then blown intermittently for 6 s more to thermalize the full sample volume while avoiding extra heating and possible polarization losses. Subsequently, the CFP was lowered inside the coil to cool down the sample and measure NMR.

After thermalization, the sample was prepared for extraction. The portable cryostat, equipped with a magnetic rail placed along the sample loading path^35^, was precooled to 4.2 K and placed close to the dDNP polarizer. The extraction procedure happened in 3 steps: firstly, the CFP was pulled up and the sample’s vial raised to the dDNP polarizer’s loading chamber without breaking the vacuum; secondly, the polarizer’s gate valve was closed and the loading chamber disconnected; thirdly, the loading chamber was manually docked at the top of the portable cryostat, its gate valve opened and the sample lowered inside the Halbach magnet. At this point NMR could be performed on the extracted sample to evaluate its relaxation properties at storage conditions. The solenoidal coil placed inside the Halbach magnet was remotely tuned and matched outside of the cryostat by means of a T/M box with two piston trimmer capacitors (Voltronics V1949). The Halbach magnet has a ^13^C resonance frequency of 10.0 MHz and 10.2 MHz at 77 K and 4.2 K, respectively. All NMR T_1_ measurements were performed using a compact bench-top spectrometer (Kea2, Magritek, Wellington, New Zealand) by applying 5° rf pulses every 5 min at 4.2 K and every 1 min at 77 K. The pulse angle was previously calibrated on a sample sending a train of 250 pulses spaced by 10 ms. Each pulse was 10 µs long and for 5 W of power. The signal decay was fit with the equation $S\left(n\right)=S\left(0\right){\cos \left(\theta \right)}^{n-1}$, where $\theta$ is the flip angle and $n$ the number of acquisitions. The software used to acquire the data was Prospa 2016 from Magritek.

### Animals handling

All animal experiments were approved by the Danish Animal Inspectorate (animal permission number 2019-15-0201-00387). Female Wistar rats (8 weeks old, weight 200 g) from Taconic Biosciences (Denmark) were included. The rats were anaesthetized with 2.5–3% sevoflurane in 2 l/min medical air. A tail vein catheter was placed for infusion of hyperpolarized glucose, and normothermia was maintained using an MRI compatible small-animal monitoring system (Small Animal Instruments Inc, USA).

### Magnetic resonance imaging with hyperpolarized Glucose

MRI was performed on a 3 T scanner (MR750, GE Healthcare, USA) with a ^13^C/^1^H rat volume coil (RAPID Biomedical, Germany). A volume of 1 ml was injected through a tail vein catheter. Anatomical images were acquired for reference. A fast spin echo sequence was used for the body (1500 ms repetition time, 11 ms echo time, 24 echo train length, 4 mm slice thickness, 160 ⨉ 160 matrix for a 160 × 160 mm field-of-view, flip angle = 16°). Hereafter, ^13^C MRSI was performed and images were acquired (65 ms repetition time, 10 × 10 matrix for a 120 × 120 mm field-of-view, spectral resolution = 1024 Hz, bandwidth = 20.000 Hz, flip angle = 10°). The transmit gain was calibrated using a phantom with appropriate load and kept constant throughout the experiment. The carbon center frequency was extrapolated from the proton frequency and kept constant within the same animal. The software used for the acquisitions was the SIGNA Platform from GE Healthcare

### Magnetic resonance imaging with hyperpolarized HP001

A 2D gradient echo spiral sequence was designed with the following parameter: FOV:120 × 120 mm, resolution = 1 × 1 mm, readout time = 21 ms, matrix size = 120 ×120. In-plane center-out 10 arms spiral trajectory was designed as variable density to minimize the ringing artifact.

^13^C dynamic angiogram images are acquired as 2D coronal view projection. The acquisition started after starting the injection. The imaging parameters were: FOV = 120 × 120 mm, resolution = 1 × 1 mm, TR/TE = 44/1 ms, flip angle = 5°, image frame time = 1/0.5 s, number of frames = 40. Only the first 16 frames are shown in the manuscript. The software used for the acquisitions was the SIGNA Platform from GE Healthcare

### Magnetic field measurements and simulations

To plot the magnetic field at the bottom panel of Fig. 3, magnetic field simulations were performed using MATLAB (MathWorks, Natick, MA, USA) and COMSOL 5.4 (COMSOL Multiphysics, Burlington, Massachusetts, USA)^31,35^. Moreover, the magnetic field along the extraction path was measured with a Hall probe Lake Shore 475 (Lake Shore Cryotronics, Westerville, OH, USA) to verify the accuracy of the simulations. The temperature profile as a function of time was used to convert the magnetic field vs distance plot to magnetic field vs time plot.

### Processing, data analysis and CAD

All data^52^ were processed and analyzed in MATLAB (MathWorks, Natick, MA, USA). All CAD drawings related to this project as well as the technical images present in Fig. 2 and Fig. 3 were realized using SolidWorks 2023 (Dassault Systèmes, Vélizy-Villacoublay, France).

### Statement of consent for publication of identifiable images from human subjects

The authors affirm that human participants provided informed consent for publication of the **Supplementary Videos 1-4**.

### Reporting summary

Further information on research design is available in the Nature Portfolio Reporting Summary linked to this article.

## Supplementary information

Supplementary Information

Description of Additional Supplementary Information

Supplementary Video 1

Supplementary Video 2

Supplementary Video 3

Supplementary Video 4

Reporting Summary

Transparent Peer Review file

## Source data

Source data

## Data Availability

All data generated in this study have been deposited in the Zenodo database under accession code zenodo.18822281 (10.5281/zenodo.18822281), and are available for download under CC BY 4.0 license. Source data are provided with this paper.

## Code availability

The MATLAB code used to analyze and process raw data has ben deposited in the Zenodo database under accession code zenodo.18822281 (10.5281/zenodo.18822281), and is available for download under CC BY 4.0 license.
