# Supplementary material for: Across-cities transportable 13C hyperpolarization using UV-induced labile radicals
Source: Nat Commun. 2026 Apr 15;17:5249. doi: 10.1038/s41467-026-71466-0 (PMC13260943; doi:10.1038/s41467-026-71466-0)
Supplement: Supplementary file 1 — Supplementary Information [file 41467_2026_71466_MOESM1_ESM.pdf]

SUPPLEMENTARY INFORMATION

Across-cities transportable ^{13}C hyperpolarization using UV-induced labile radicals

Andrea Capozzi [1,2] *, Magnus Karlsson [2], Yupeng Zhao [2], Jan Kilund [2], Esben Søvsø Szocska Hansen [3], Lotte Bonde Bertelsen [3], Christoffer Laustsen [3], Jan Henrik Ardenkjær-Larsen [2], and Mathilde H. Lerche [2].

[1] *LIFMET, Department of Physics, EPFL, Station 6 (Batiment CH), 1015 Lausanne (Switzerland).*

[2] *HYPERMAG, Department of Health Technology, Technical University of Denmark, Building 349, 2800 Kgs Lyngby (Denmark).*

[3] *The MR Center, Department of Clinical Medicine, Aarhus University, Palle Juul-Jensens Boulevard 99, 8200 Aarhus N (Denmark)*

Corresponding author

*Dr. Andrea Capozzi

EPFL SB IPHYS LIFMET

CH F0 632 (Bâtiment CH), Station 6, CH-1015 Lausanne

Email: andrea.capozzi@epfl.ch; ORCID: 0000-0002-2306-9049

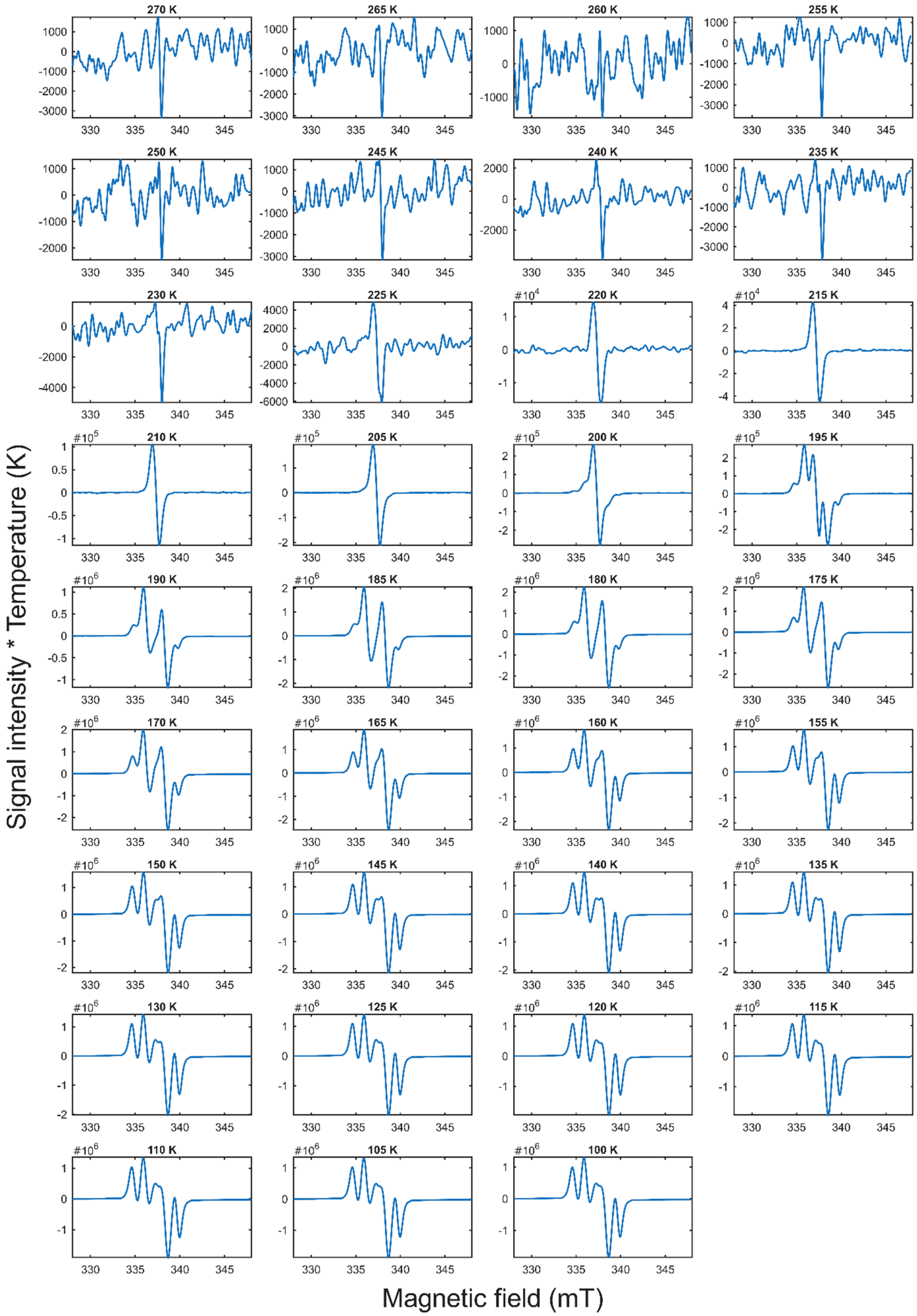
270 K
265 K
260 K
255 K
250 K
245 K
240 K
235 K
230 K
225 K
220 K
215 K
210 K
205 K
200 K
195 K
190 K
185 K
180 K
175 K
170 K
165 K
160 K
155 K
150 K
145 K
140 K
135 K
130 K
125 K
120 K
115 K
110 K
105 K
100 K
#10^4
#10^5
#10^6
Signal intensity * Temperature (K)
Magnetic field (mT)

Figure S1. Radical quench dynamics for alpha-ketoglutaric acid (AKG): one 10 μL bead of 2M glucose and 0.7 M AKG dissolved in d_8-glycerol:D_2O 1:1 (v/v) was irradiated for 300 s vial UV-light at 35 W/cm^2 and transferred in the VTI of an X-band Electron Spin Resonance (ESR) spectrometer. The temperature was increased in steps of 5 K from 100.15 K to 275.15 K (n=1). At each temperature step the ESR signal was acquired. No signal could be detected above 230.15 K, thus, 225.15 K was set as heating threshold for the thermalization experiment. Source data are provided as a Source Data file.

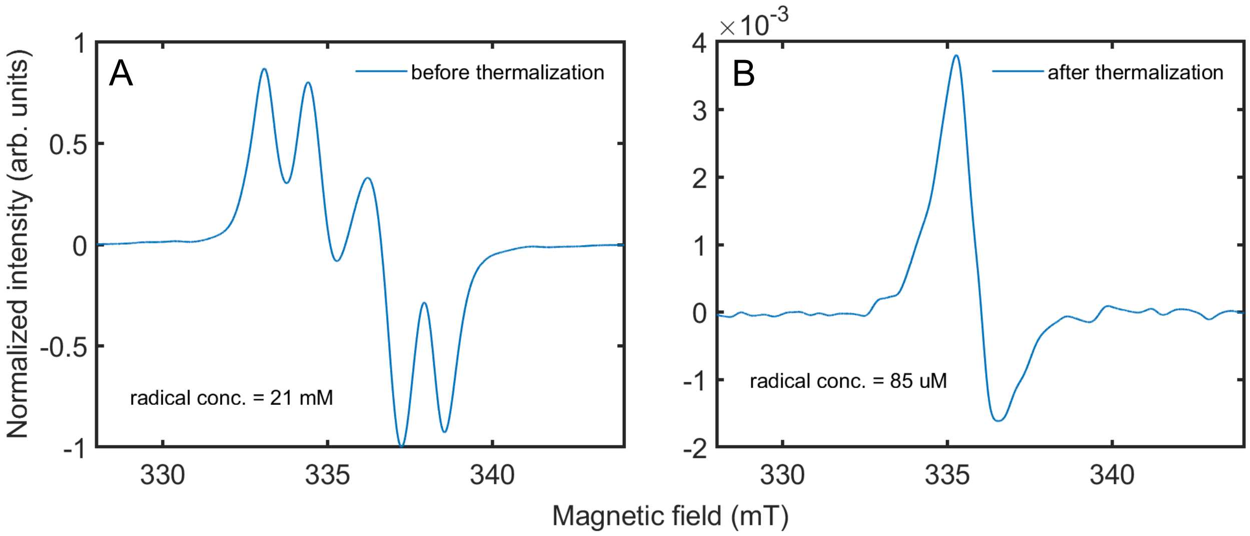

Figure S2. Radical left-over after thermalization: ESR signal of the glucose sample before the DNP experiment (n=1) (**A**). ESR signal of the glucose sample after thermalization (n=1) (**B**); the heating process by means of He gas quenched more that 99.5% of the initial radical concentration. Source data are provided as a Source Data file.

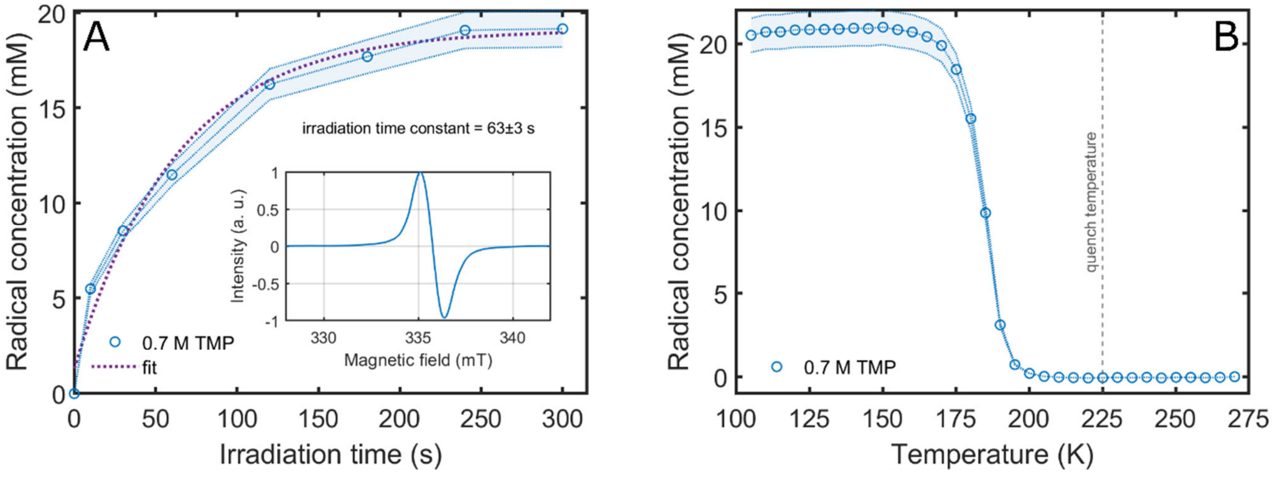
A
Radical concentration (mM)
Irradiation time (s)
irradiation time constant = 63±3 s
Intensity (a. u.)
Magnetic field (mT)
0.7 M TMP
fit
B
Radical concentration (mM)
Temperature (K)
quench temperature
0.7 M TMP

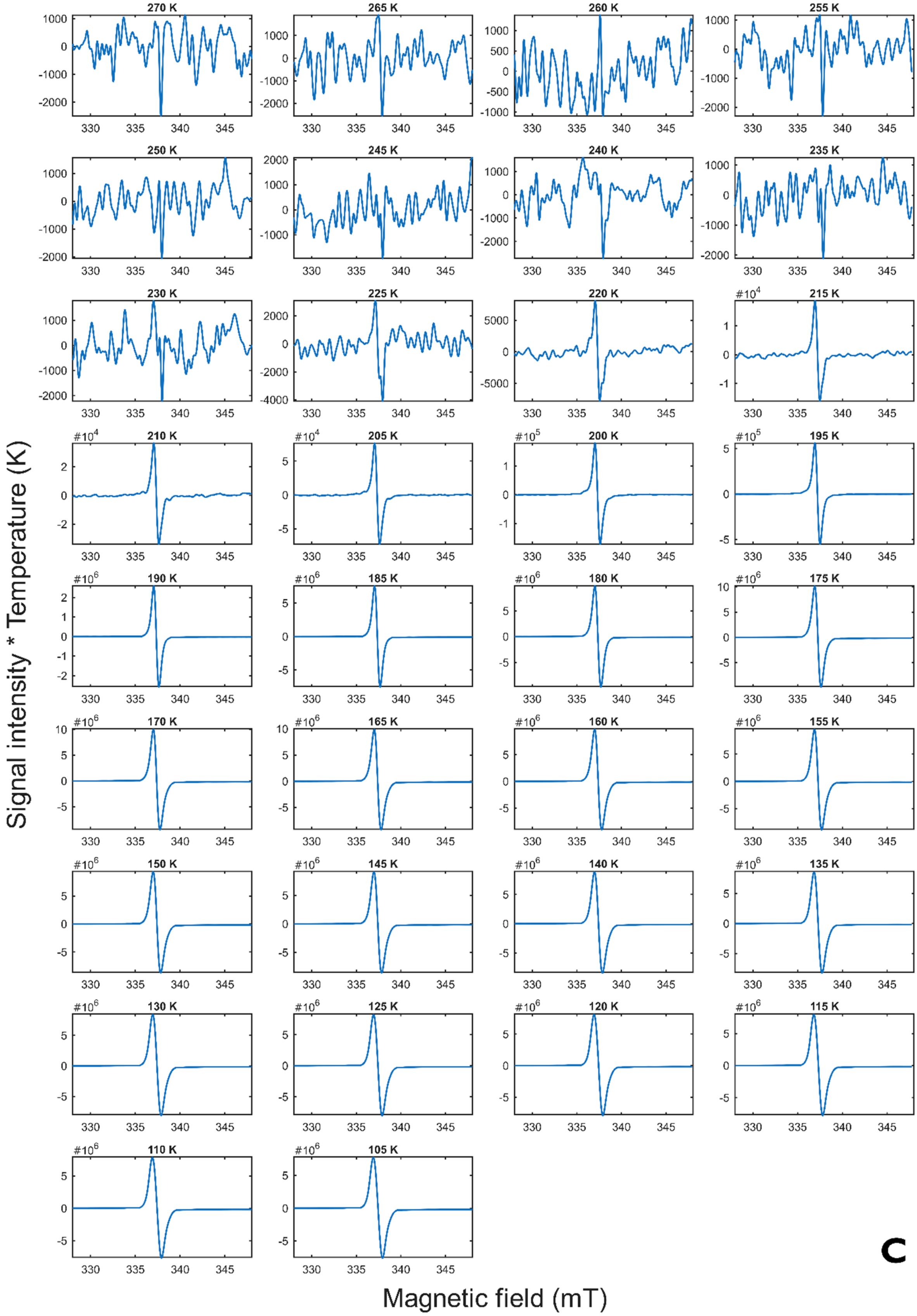
270 K
265 K
260 K
255 K
250 K
245 K
240 K
235 K
230 K
225 K
220 K
215 K
210 K
205 K
200 K
195 K
190 K
185 K
180 K
175 K
170 K
165 K
160 K
155 K
150 K
145 K
140 K
135 K
130 K
125 K
120 K
115 K
110 K
105 K
Signal intensity * Temperature (K)
Magnetic field (mT)
C

Figure S3. Radical properties of the glucose sample prepared with trimethylpyruvic acid (TMP) as radical precursor: The sample contains 2M glucose and 0.7 M TMP dissolved in glycerol:water 1:1 (v/v). Figure's panels description follows in the order.

Radical generation by means of UV-light irradiation in liquid nitrogen measured with X-band ESR (n=1); the error band (light blue area) is +/- 5% of the measured radical concentration of each data point and comes from spectrometer calibration; a mono-exponential curve (violet dotted line) was fitted to the data, its mathematical expression is $C(t) = C_\infty\left(1 - exp(-t/T_i)\right)$, where t, T_i, C_∞, $C(t)$ represent the time of irradiation, the irradiation time constant, the concentration at saturation, and the concentration at time t, respectively. T_i was 63±3 s with R^2 = 0.95 (**A**). Radical concentration as a function of temperature measured with X-band ESR (n=1); the error band (light blue area) is +/- 5% of the measured radical concentration of each data point and comes from spectrometer calibration (**B**). Quench temperature dynamic of the sample (n=1) (**C**). Source data are provided as a Source Data file.

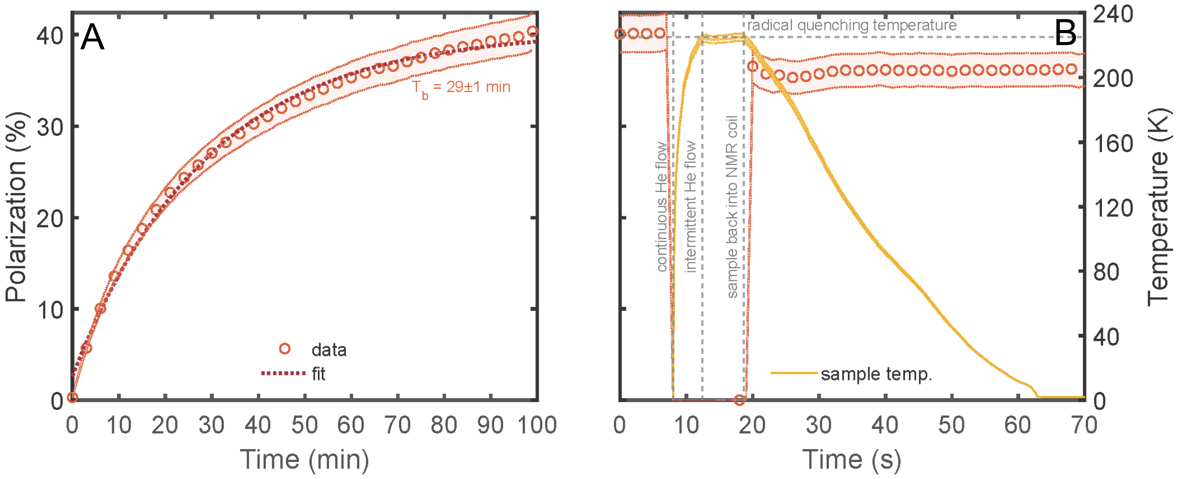

Figure S4. Effect of a protonated matrix on Dynamic Nuclear Polarization (DNP) performance: Polarization (n=1) (**A**) and thermalization (n=1) (**B**) for a glucose sample prepared with a protonated solvent and AKG as radical precursor. Deuteration of the solvent, increased the maximum achievable polarization by 10%; deuteration had no effect on the behavior of the sample during thermalization. In **panel A** a mono-exponential curve (violet dotted line) was fitted to the data, its mathematical expression is $P(t) = P_\infty\left(1 - exp(-t/T_b)\right)$, where t, T_b, P_∞, $P(t)$ represent the time of polarization, the polarization time constant, the polarization at infinite time, and the polarization at time t, respectively. T_b was 29±1 min with R^2 = 0.97. In **panel B** the sample temperature as a function of time is also reported (yellow curve). In both panels, the error band (light red area) is +/- 5% of the measured polarization value of each data point and comes from NMR spectrometer calibration. Source data are provided as a Source Data file.

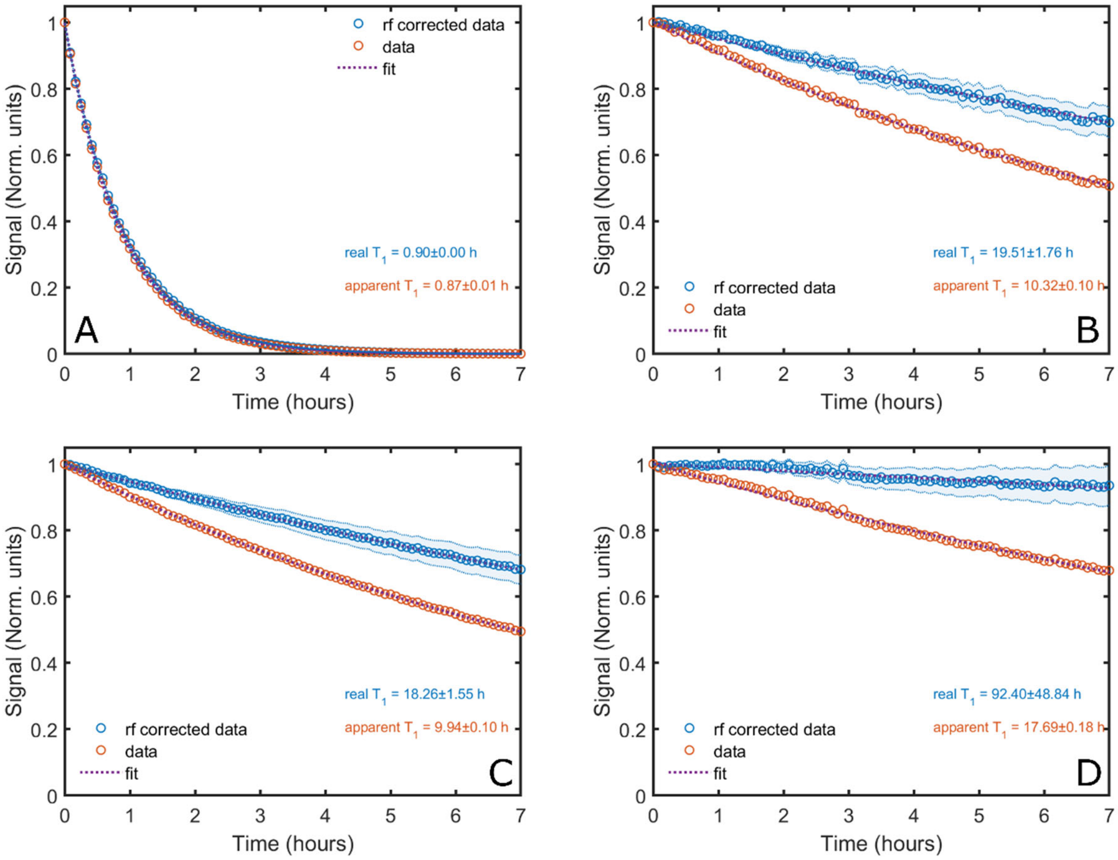

Figure S5. Relaxation of different sample preparations at transport conditions: Example of T_1 relaxation at 4 K and 1 T measured by means of 5° excitation pulses every 5 min for hyperpolarized and thermalized glucose samples prepared with TMP as radical precursor and a protonated solvent (n=1) (**A**); dTMP as radical precursor and a protonated solvent (n=1) (**B**); AKG as radical precursor and a protonated solvent (n=1) (**C**); AKG as radical precursor and a deuterated solvent (n=1) (**D**). In all panels, the red circles represent the integral of the spectrum measured from the spectrometer; the blue circles were corrected for the rf pulse. The blue shaded areas represent the error on the T_1 values coming from the error on the estimation of the flip angle. A mono-exponential curve (violet dotted line) was fitted to the data, its mathematical expression is $P(t) = P_0\left(exp(-t/T_1)\right)$, where t, T_1, P_0, $P(t)$ represent the time of polarization, the relaxation time constant, the polarization at time zero, and the polarization at time t, respectively. Source data are provided as a Source Data file.

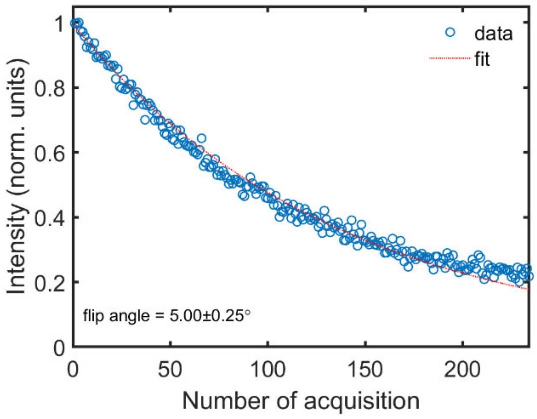

Figure S6. Portable cryostat B_1 calibration on ^{13}C: One hyperpolarized and thermalized glucose sample was used to calibrate the pulse angle of the NMR probe of the transportable cryostat. A train of 250 pulses spaced by 10 ms and with a length = 10 µs and a power = 5 W made the signal to decay (n=1). The decay was fitted with the equation $S(n) = S(0)cos(\theta)^{n-1}$, where θ is the flip angle and n the number of acquisitions. Knowing that $\theta = \gamma_{13C}B_1\tau$, where γ_{13C}, B_1, and τ are the carbon gyromagnetic ration, the magnetic field intensity generated by the NMR coil and the length of the pulse, respectively, $B_1 = 1.30 \pm 0.06\ G$. Source data are provided as a Source Data file.

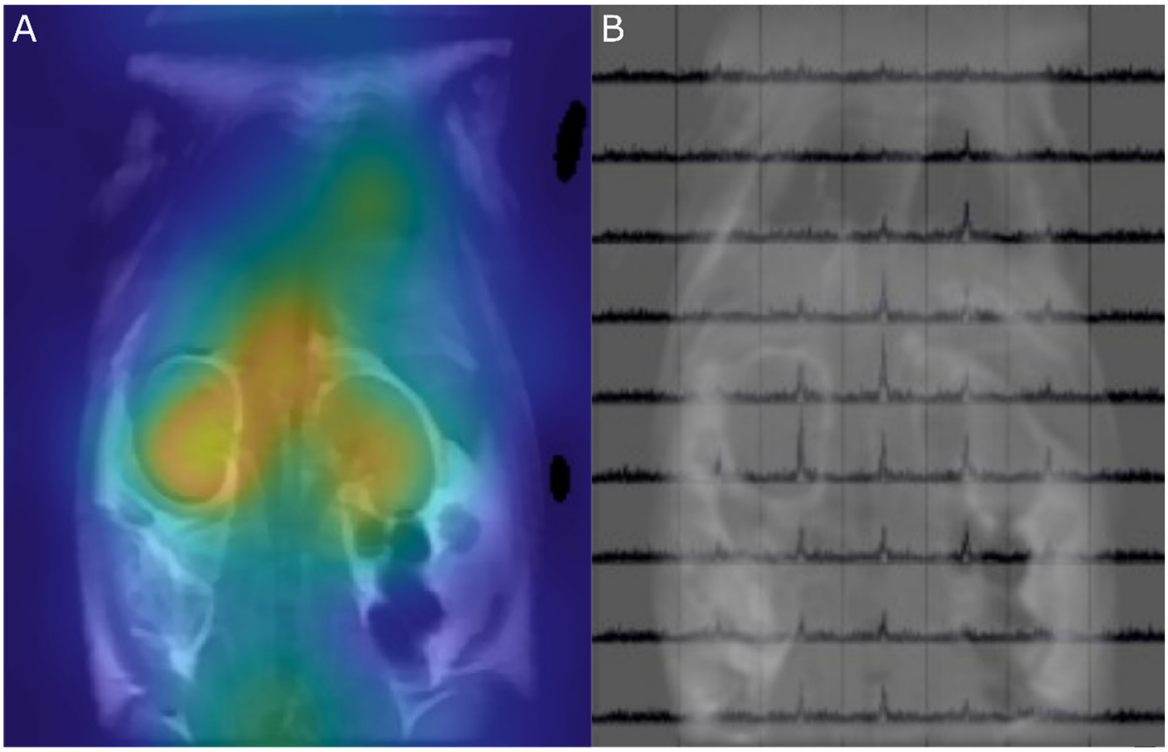

Figure S7. Chemical Shift Imaging (CSI) of glucose in a healthy rat model: MRI was performed on a 3T scanner (MR750, GE Healthcare, USA) with a $^{13}C/^{1}H$ rat volume coil (RAPID Biomedical, Germany). A volume of 1 ml was injected through a tail vein catheter. Anatomical images were acquired for reference. A fast spin echo sequence was used for the body (1500 ms repetition time, 11 ms echo time, 24 echo train length, 4 mm slice thickness, 160 × 160 matrix for an 160 x 160 mm field-of-view, flip angle = 16°). Hereafter, ^{13}C CSI was performed and images were acquired (65 ms repetition time, 10 x 10 matrix for a 120 x 120 mm field-of-view, spectral resolution = 1024 Hz, bandwidth = 20.000 Hz, flip angle = 10°). The transmit gain was calibrated using a phantom with appropriate load and kept constant throughout the experiment. The carbon center frequency was extrapolated from the proton frequency and kept constant within the same animal (n=1).

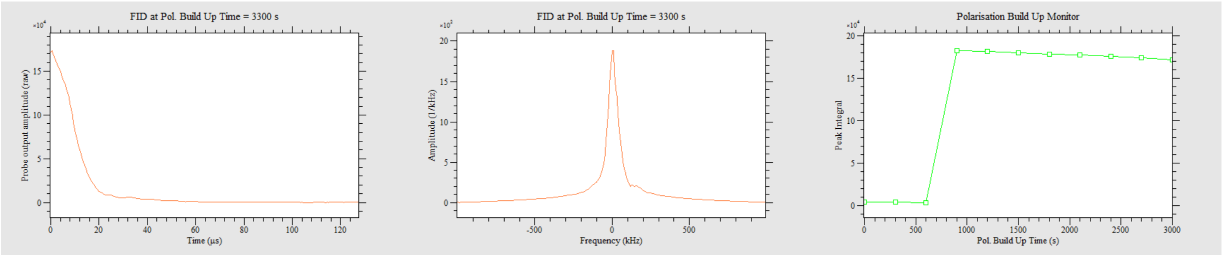

Figure S8. Signal check after extraction of the HP001 sample: Before attempting transport to Aarhus University Hospital, the signal of the extracted sample was first checked and compared to previous experiments. We report here

a print screen of the spectrometer before disconnecting the electronics of the transportable cryostat. The three panel, from left to right, represent the signal FID, its Fourier Transform (NMR spectrum), and the integral of the spectrum as a function of time. Points were acquired every 5 min. The acquisition was started before insertion of the sample into the cryostat to get a base line reference and evaluate the decay. Before departure, the signal was observed for 45 min and negligible signal loss detected (n=1). During transport, no NMR pulsing was applied to conserve as much signal as possible. The image is a print screen from the spectrometer.

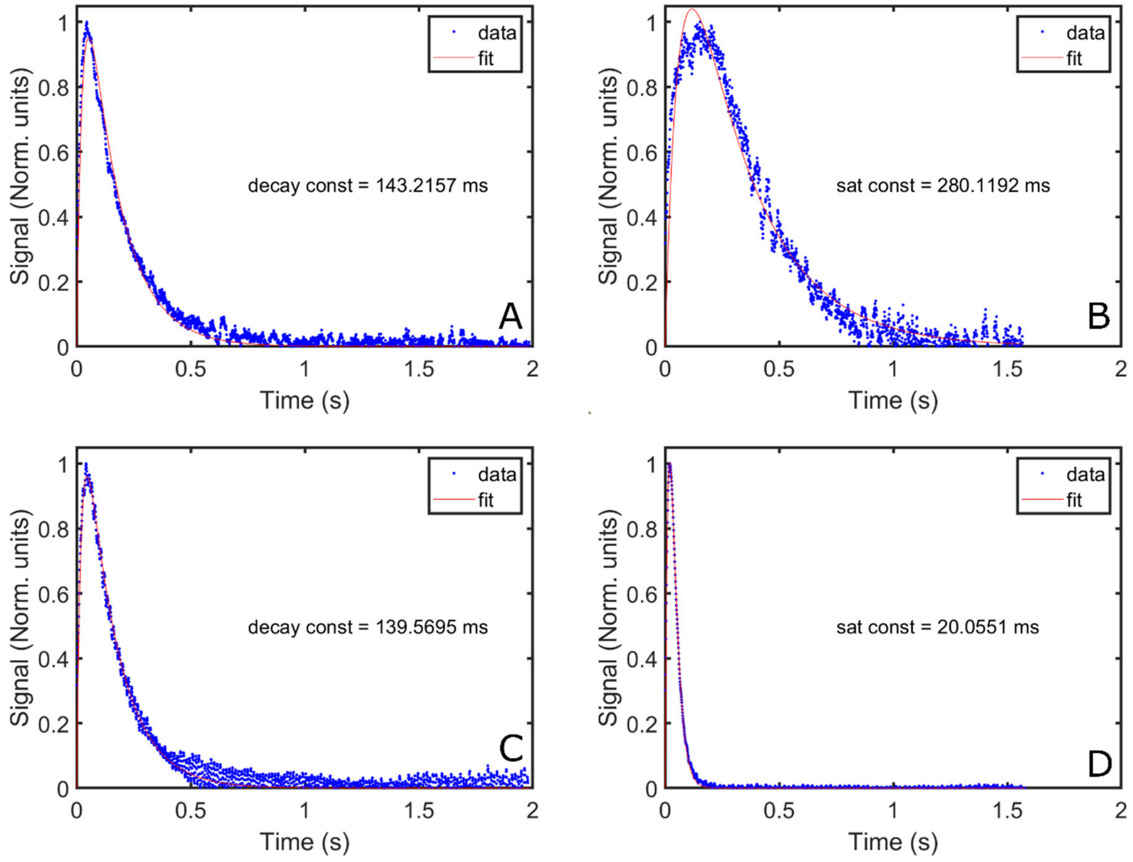

Figure S9. Effect of microwave Frequency Modulation (FM) on electron excitation and relaxation: Example of (n=1) electron spins dynamics measured by means of LOD-ESR [1], of the radicals induced on a sample containing 2 M glucose and 0.7 M AKG in a deuterated solvent upon switching OFF the microwaves after excitation with no FM (**A**); switching ON the microwaves after relaxation with no FM (**B**); switching OFF of the microwaves after excitation with 50 MHz FM (**A**); switching ON of the microwaves after relaxation with 50 MHz FM (**D**). While relaxation was FM insensitive, excitation became much faster when FM was applied. Extraction of the relaxation and excitation time constants *(T_c)* were performed by fitting the signal evolution in the time domain to the equation $S(t) = A\left(exp(-t/T_c) - exp(-t/\tau)\right)$ where A is a proportionality factor depending on the sample properties and measuring parameters and τ the time constant of the measuring split solenoid coil. Source data are provided as a Source Data file.

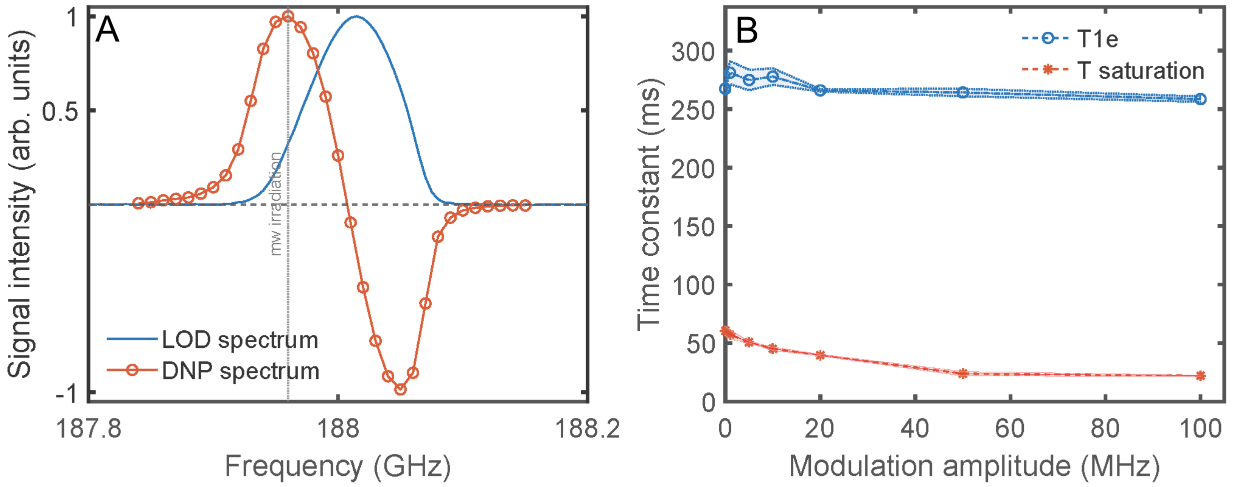

Figure S10. LOD-ESR and DNP characterization of a 2M glucose sample doped with 20 mM of trityl radical: as a comparison (n=1) we report the LOD-ESR and DNP investigation of a sample that did not benefit from FM (i.e. no increase in DNP enhancement). The ESR spectrum and DNP spectrum are overlapped in **panel A** and the grey line represents the microwave frequency (187.96 GHz) that optimizes the nuclear signal enhancement. The relaxation and excitation dynamics of the radical spins as a function of FM is reported in **panel B** and were measured at optimal microwave conditions. It is important noticing that, differently from the AKG sample, excitation was much faster than relaxation already for monochromatic microwave irradiation; the error bands (light blue area for spin relaxation measurements and light red area for spin excitation measurements) comes from the accuracy of the fit for each measurement (see Fig. S9 as an example of single acquisition and fit). Source data are provided as a Source Data file.

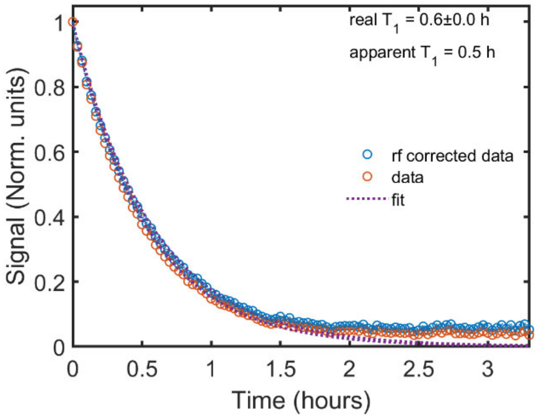

Figure S11. Glucose sample relaxation at 1 T and 77 K: The relaxation of the sample (n=1) prepared with 2M glucose and 0.7 AKG dissolved in a deuterated solvent is reported after thermalization and transfer to the transportable cryostat filled with liquid nitrogen (77 K). A mono-exponential curve (violet dotted line) was fitted to the data, its mathematical expression is $P(t) = P_0\left(exp(-t/T_1)\right)$, where t, T_1, P_0, $P(t)$ represent the time of polarization, the

relaxation time constant, the polarization at time zero, and the polarization at time t, respectively. Source data are provided as a Source Data file.

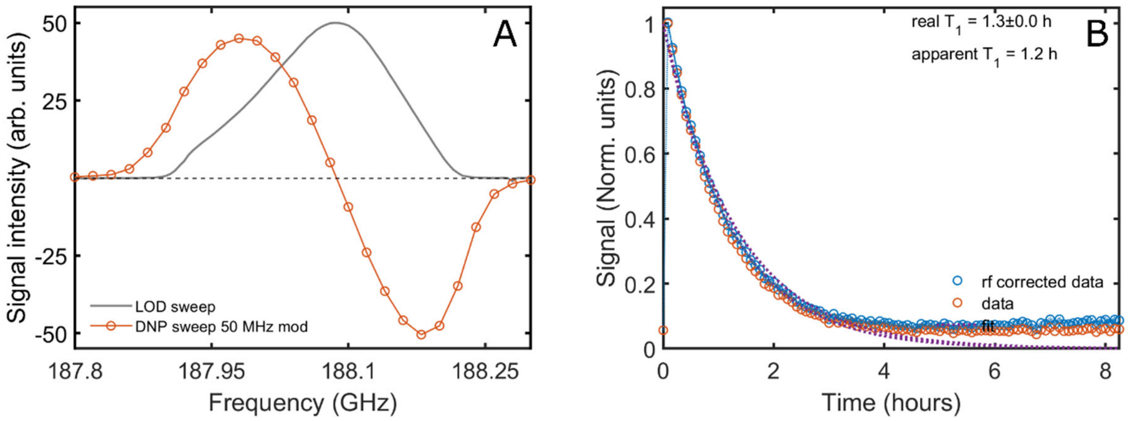

Figure S12. Note on pyruvic acid: The DNP performance from the polarizer (**A**) and relaxation from the transportable cryostat,1 T/4.2 K, after thermalization (**B**) are reported for a UV-irradiated sample prepared with 7 M [d_4, 1-^{13}C]pyruvic acid dissolved in d_8-glycerol:D_2O (n=1). **Panel A** shows the overlap of the DNP spectrum to the Lod spectrum. In **panel B** a mono-exponential curve (violet dotted line) was fitted to the data, its mathematical expression is $P(t) = P_0\left(exp(-t/T_1)\right)$, where t, T_1, P_0, $P(t)$ represent the time of polarization, the relaxation time constant, the polarization at time zero, and the polarization at time t, respectively. Source data are provided as a Source Data file.

Supplementary References

1. Capozzi, A., Karlsson, M., Petersen, J. R., Lerche, M. H. & Ardenkjaer-Larsen, J. H. Liquid-State ^{13}C Polarization of 30% through Photoinduced Nonpersistent Radicals. *J. Phys. Chem. C* **122**, 7432–7443 (2018).
