# Supplementary material for: Across-cities transportable 13C hyperpolarization using UV-induced labile radicals
Source: Nat Commun. 2026 Apr 15;17:5249. doi: 10.1038/s41467-026-71466-0 (PMC13260943; doi:10.1038/s41467-026-71466-0)
Supplement: Supplementary file 2 — Description of Additional Supplementary Information [file 41467_2026_71466_MOESM2_ESM.pdf]

Description of Additional Supplementary Files

File Name: Supplementary Video 1
Description: In this video, we show the experimental procedure and equipment to increase, inside the dDNP polarizer, the sample temperature above the radicals' quench point.

File Name: Supplementary Video 2
Description: In this video, we show the sample extraction from the dDNP polarizer and storage inside the transportation device. Solid-state signal was checked after extraction via NMR.

File Name: Supplementary Video 3
Description: In this video, we show the ease of use and portability of the transportation device.

File Name: Supplementary Video 4
Description: In this video, we show the dissolution and injection of a hyperpolarized 13C-MRI contrast agent, produced off-site and transported to the place of use.
