# Supplementary material for: Across-cities transportable 13C hyperpolarization using UV-induced labile radicals
Source: Nat Commun. 2026 Apr 15;17:5249. doi: 10.1038/s41467-026-71466-0 (PMC13260943; doi:10.1038/s41467-026-71466-0)
Supplement: Supplementary file 7 — Reporting Summary [file 41467_2026_71466_MOESM7_ESM.pdf]

nature portfolio

Corresponding author(s): Andrea Capozzi

Last updated by author(s): Mar 1, 2026

Reporting Summary

Nature Portfolio wishes to improve the reproducibility of the work that we publish. This form provides structure for consistency and transparency in reporting. For further information on Nature Portfolio policies, see our Editorial Policies and the Editorial Policy Checklist.

Statistics

For all statistical analyses, confirm that the following items are present in the figure legend, table legend, main text, or Methods section.

n/a	Confirmed	
☐	☒	The exact sample size (n) for each experimental group/condition, given as a discrete number and unit of measurement
☐	☒	A statement on whether measurements were taken from distinct samples or whether the same sample was measured repeatedly
☒	☐	The statistical test(s) used AND whether they are one- or two-sided<br>*Only common tests should be described solely by name; describe more complex techniques in the Methods section.*
☒	☐	A description of all covariates tested
☒	☐	A description of any assumptions or corrections, such as tests of normality and adjustment for multiple comparisons
☐	☒	A full description of the statistical parameters including central tendency (e.g. means) or other basic estimates (e.g. regression coefficient) AND variation (e.g. standard deviation) or associated estimates of uncertainty (e.g. confidence intervals)
☒	☐	For null hypothesis testing, the test statistic (e.g. F, t, r) with confidence intervals, effect sizes, degrees of freedom and P value noted<br>*Give P values as exact values whenever suitable.*
☒	☐	For Bayesian analysis, information on the choice of priors and Markov chain Monte Carlo settings
☒	☐	For hierarchical and complex designs, identification of the appropriate level for tests and full reporting of outcomes
☒	☐	Estimates of effect sizes (e.g. Cohen's d, Pearson's r), indicating how they were calculated

Our web collection on statistics for biologists contains articles on many of the points above.

Software and code

Policy information about availability of computer code

Data collection	NMR data from DNP polarizer were collected using proprietary software VNMRJ 4.2 from Agilent Technology; NMR data from transportable cryostat were collected using the proprietary software Prospa 2016 from Magritek; X-band ESR data were collected using the proprietary software ESR Studio from Magnettech; high-field ESR data were collected with home made software programmed in LabView 2019 (National Instruments); MR images were collected using the proprietary SIGNA Platform from GE Healthcare; All CAD drawings related to this project as well as the technical images present in Figure 2 and Figure 3 were realized using SolidWorks 2023 (Dassault Systèmes, Vélizy-Villacoublay, France). Magnetic field simulations were performed using MATLAB 2023 (MathWorks, Natick, MA, USA) and COMSOL 5.4 (COMSOL Multiphysics, Burlington, Massachusetts, USA)
Data analysis	All data were processed and analyzed in MATLAB 2023 (MathWorks, Natick, MA, USA). The MATLAB code used to analyze and process raw data has ben deposited in the Zenodo database under accession code zenodo.18822281 (https://doi.org/10.5281/zenodo.18822281), and is available for download under CC BY 4.0 license

For manuscripts utilizing custom algorithms or software that are central to the research but not yet described in published literature, software must be made available to editors and reviewers. We strongly encourage code deposition in a community repository (e.g. GitHub). See the Nature Portfolio guidelines for submitting code & software for further information.

Data

Policy information about availability of data

All manuscripts must include a data availability statement. This statement should provide the following information, where applicable:

- Accession codes, unique identifiers, or web links for publicly available datasets
- A description of any restrictions on data availability
- For clinical datasets or third party data, please ensure that the statement adheres to our policy

All data generated in this study have been deposited in the Zenodo database under accession code zenodo.18822281 (https://doi.org/10.5281/zenodo.18822281), and are available for download under CC BY 4.0 license. Source Data are provided with this paper.

Research involving human participants, their data, or biological material

Policy information about studies with human participants or human data. See also policy information about sex, gender (identity/presentation), and sexual orientation and race, ethnicity and racism.

Reporting on sex and gender	n.a.
Reporting on race, ethnicity, or other socially relevant groupings	n.a.
Population characteristics	n.a.
Recruitment	n.a.
Ethics oversight	n.a.

Note that full information on the approval of the study protocol must also be provided in the manuscript.

Field-specific reporting

Please select the one below that is the best fit for your research. If you are not sure, read the appropriate sections before making your selection.

☒ Life sciences ☐ Behavioural & social sciences ☐ Ecological, evolutionary & environmental sciences

For a reference copy of the document with all sections, see nature.com/documents/nr-reporting-summary-flat.pdf

Life sciences study design

All studies must disclose on these points even when the disclosure is negative.

Sample size	No sample size calculation was performed. In vivo MRI acquisition was performed as final test and at proof of concept level. No particular pathology was investigated. All animals were healthy subjects. The study entailed the employment of 3 (three) rodents in total. One was involved for the first experiment to asses SNR threshold. In the second in vivo experiment 2 more rodents were involved in the study: the first was injected using a contrast agent transported and produced off-site; the second, as control, was injected with the same contrast agent produced on-site.
Data exclusions	no in-vivo data were excluded from this study
Replication	Reproducibility of the experimental findings were limited to the question: "can we see signal in vivo". To this extent, all experiments were successful.
Randomization	Randomization was not relevant to our study. No groups were established. We worked with three living subjects only. No statistical analysis was performed for the in vivo experiments.
Blinding	Blinding was not relevant to this study because no groups were established. We worked with three living subjects only. No statistical analysis was performed for the in vivo experiments.

Reporting for specific materials, systems and methods

We require information from authors about some types of materials, experimental systems and methods used in many studies. Here, indicate whether each material, system or method listed is relevant to your study. If you are not sure if a list item applies to your research, read the appropriate section before selecting a response.

Materials & experimental systems

n/a	Involved in the study
[x]	[ ] Antibodies
[x]	[ ] Eukaryotic cell lines
[x]	[ ] Palaeontology and archaeology
[ ]	[x] Animals and other organisms
[x]	[ ] Clinical data
[x]	[ ] Dual use research of concern
[x]	[ ] Plants

Methods

n/a	Involved in the study
[x]	[ ] ChIP-seq
[x]	[ ] Flow cytometry
[x]	[ ] MRI-based neuroimaging

Animals and other research organisms

Policy information about studies involving animals; ARRIVE guidelines recommended for reporting animal research, and Sex and Gender in Research

Laboratory animals	This study involved Female Wistar rats (8 weeks old, weight 200g) from Taconic Biosciences (Denmark)
Wild animals	Not involved in this study
Reporting on sex	Sex was not considered in this study and sex-based analysis were not performed. Our findings are not related to sex. Animals were involved only to asses in vivo snr in MR images "in real conditions". The latter has nothing to do with sex.
Field-collected samples	The study did not involve samples collected from the field
Ethics oversight	All animal experiments were approved by the Danish Animal Inspectorate (animal permission number 2019-15-0201-00387)

Note that full information on the approval of the study protocol must also be provided in the manuscript.

Plants

Seed stocks	n.a.
Novel plant genotypes	n.a.
Authentication	n.a.
