# Supplementary material for: Across-cities transportable 13C hyperpolarization using UV-induced labile radicals
Source: Nat Commun. 2026 Apr 15;17:5249. doi: 10.1038/s41467-026-71466-0 (PMC13260943; doi:10.1038/s41467-026-71466-0)
Supplement: Supplementary file 8 — Transparent Peer Review file [file 41467_2026_71466_MOESM8_ESM.pdf]

nature portfolio

Peer Review File

Across-cities transportable 13C hyperpolarization using UV-induced labile radicals

Corresponding Author: Dr Andrea Capozzi

This file contains all reviewer reports in order by version, followed by all author rebuttals in order by version.

Version 0:

Reviewer comments:

Reviewer #1

(Remarks to the Author)
This work represents a clear and valuable continuation of the study led by Capozzi and Ardenkjær-Larsen. Building on their previous advancements in small dDNP systems, photo-induced radicals, and hyperpolarization storage units, the authors now demonstrate a proof-of-principle transport of 13C hyperpolarization between cities. Similar hyperpolarization transport experiments have previously been performed with xenon, and the concept of such transportation for 13C-labeled molecules was, among others, discussed by Kempf et al. in JACS (2015).
A particularly notable strength of this study is the integration of multiple technical advancements in hyperpolarization, which together enabled approximately five hours of transport with only ~50% polarization loss—an impressive achievement. It is unfortunate that the concept could not be immediately used for pyruvate-like molecules due to rapid relaxation.
I recommend this work for publication. However, the current version of the figures is of low visual quality, and some font sizes are too small to read comfortably. I hope this will be addressed in the final version. Furthermore, despite the authors' efforts, the manuscript still contains some jargon that is not always self-explanatory, with definitions only found in the Methods section. To improve accessibility for a broader readership, I strongly encourage a critical revision of both the text and the figures.
Lastly, the statement: "Raw data are available from the corresponding author (andrea.capozzi@epfl.ch) on reasonable request." is no longer acceptable. Raw data must be deposited in publicly accessible repositories supported by Nature Publishing, national, or European open science initiatives. This practice is now a standard requirement and reflects good scientific conduct.

Some other comments.
The introduction is nice; however, it does not discuss Xenon hyperpolarization. I see that you are formally right, that here is the first 13C MRI experiment; however, it is not the first transport of hyper in humans in general, and not the longest.

146-147 "The high field ESR spectrum, measured by means of a LOngitudinal Detection technique (LOD-ESR)" – It would be helpful if you could explain what this is. You are doing this for the DNP spectrum, and it helps to streamline the text.

180-181 "No further decrease was observed for higher values of FM." This statement is not clear to me, as I do see the decline in Figure 1D. Why are the error bars so different for blue and red?

Figure 1: Some text elements are too small. The green arrow does not have an immediate visual explanation; maybe you can design it with a thick tail instead of connecting it to the angle.

202: (panel A) – Figure 2A?, and below for B

214 – "the magnetically enforced probe of the polarizer" – please explain the meaning of this
221- "Halbach array along the polarizer probe" – what is the polarizer probe? MW or NMR coil? Does not make sense. And I do not see it on the figure.
Fig 3: please assigne all relevant components: the gate valve, dynamic seal, the magnetically enforced probe, the gate valve of the transportation, the magnetically enforced probe of the portable cryostat, and others
In general although this figure probably adequate it does not help to a reader who did not design the system and not familiar

with the jargon used in the lab.
317-318 “The achieved level of contrast in the images was not far from what was obtained from a standard trityl radical-doped sample polarized on-site.” – is it reported? Could be reminded here, but it should be at least a factor of 2 different.

337 - “Getting rid of methyl group relaxation, via deuteration (i.e. dTMP)” – can you comment on how deuteration helps? I assume relaxation via tunneling should also be in the deuterated case.

338 – “After thermalization, the abundant nuclear species in our samples (i.e. 1H and 2H) are completely depolarized because of fast spin diffusion during heating” – How? If all nuclei are hyperpolarized, then diffusion should not be so important here unless there is a rapid relaxation on its own.

404 - HP001 was it also purchased in S-A? Would be helpful to have a catalog numbers for all commertial products that readers can reproduce and have ideas about purity of the used compounds.

What about trytil? Was it also purchased in S-A?

“quartz Dewar (Magnettech” – catalog id?

“The CFP is built using two concentric tubes (an outer lumen - black - and an inner lumen - red -
426 ending with a nozzle - green-)” – Do you refer to any figure? Otherwise, not sure what the colors could mean.

Reviewer #2

(Remarks to the Author)
Nature Communications manuscript 25-29843 by Capozzi et al describes the generation of long-lasting 13C hyperpolarization using quenchable UV-induced radicals. The relatively stable 13C hyperpolarization, with T1s in excess of 10 hours, allows for off-site centralized production followed by transportation to and use by a remote study site. A successful demonstration was given for production and utilization between two sites 5 hours and 320 km apart.

As also indicated by the authors themselves, this is highly analogous to the centralized production of 18F-FDG for PET imaging and eliminates, for 13C hyperpolarization, the need for very expensive hyperpolarization equipment. This is a major step forward in the more routine and widespread use of 13C hyperpolarization methodology.

Overall, the work is described clearly and with sufficient detail, such that I can recommend publication with only minor suggestions.

Specific comments:
1. The text on top of Page 5 in the Introduction is a bit confusing. It is stated that for traditional EPAs “they must be produced on site because of their minute-long half-life in the liquid state”. However, this is not really the relevant part as the half-life of any hyperpolarization in the liquid-state is short-lived independent of the radical used. A few sentences later, the authors make the relevant statement themselves stating “they would destroy the MCAs’ nuclear spin order within few milliseconds when, during extraction from the polarizer, the frozen HP sample is exposed to low magnetic field.” I think this entire section can be rephrased more clearly, stating the relevant considerations for both traditional and UV-induced radicals.
2. Page 15 – For consistency with earlier text in the Introduction, use MRSI instead of CSI. There is no need to use 2 acronyms for the same technique.
3. Figure 5 - This Figure can benefit from text within the panels. Instead of fully relying on text within the legend, add labels like 'trityl-DNP' and 'UV-DNP'. Add time stamps to the dynamic series. Generally, it is good to be able to understand the content of a figure without having to read the legend.
4. Page 21, line 393 - … it was earlier demonstrated that changing …

Reviewer #3

(Remarks to the Author)
I started reading the paper with great interest as transportation of HP 13C agents would be a real game-changer for the field, and for the application of the technique in clinical practice. Hence, I was a little disappointed seeing that only perfusion HP agent was successfully used (with 50% polarization, loss). While this is still a very nice result, HP 13C will not make it to clinical use thanks to perfusion scans. There are plenty of other options already available for that. It is the metabolic imaging possibilities of HP 13C (that the authors describe in the rather long introduction) that are of real clinical interest. Unfortunately, the metabolic imaging experiment tried in this study did not work. To achieve a real impact, this needs to change.

Main points:
This work is an extension of the authors work in transporting UV polarized agents (refs 36 and 41) with the use of new

transport device (ref 40) to allow “across-cities” transport. The distance over which the polarization was transported being the main (and likely only) novelty. As stated above, this could still be considered exciting, should you be able to demonstrate a successful metabolic experiment. Please consider making the trip again to deliver this essential piece of data.

The fact that you have used a “clinical” scanner is hardly of importance given you are still scanning a rat using a substrate not allowed for clinical use. Please don’t try to oversell the work.

You explain that error in corrected T1s came from calibration of B1, while there is definitely an effect, it is more likely that you are not able to properly fit such long T1s with your current experiment (only up to 7h readout). Also, your claim that everything is fine up to a non-corrected T1 below 10h, does not hold as your experiments shown that dTMP with 10.3h T1 still has a negligible error. Please don’t make up cut-offs without any evidence.

In the discussion section, you try to balance out the fact that you lost 50% polarization during transport by promoting UV induced polarization without persistent radicals in the solution. While this is indeed a nice feature of these solutions, the benefits of this polarization technique has been discussed elsewhere, Plus it only makes your case worse as what you are saying basically is “If we made these on site we could have skipped the QC and injected faster”, hence loosing even less polarization in comparison to the transported sample. Or do you want to say that your sample had to go through QC procedure at the recipient site, which was probably not necessary and could have saved you some polarization? If yes, state that rather than general stories. Also, would it be possible to use polarization by UV induced radicals for both transported and non-transported samples?

Talking about stories, the whole manuscript reads more like an adventure story than a scientific work, please consider rewriting.

One minor point. For better comparison between the two perfusion experiments, I would recommend dropping the first “pre-perfusion” frame.

Reviewer #4

(Remarks to the Author)
Overall the authors have made an interesting and innovative contribution to the field of MRSI. The methodology does feature some cumbersome elements, such as the lengthy transportaion, but it is a step towards democratisation of MRSI and they do contribute to increased sensitivity. Overall the manuscript does contribute to the scientific body of literature and is an adequate proof of concept work.

Minor comments attached to the documents as comments.

Version 1:

Reviewer comments:

Reviewer #1

(Remarks to the Author)
This is very nice proof-of-concept work that fits the journal perfectly. I recommend it with minor corrections for publications.

96-97 lines read as if point 1 (line 89) has an advantage over point 2 (line 92) because it is readily biocompatible. It feels that polymers could be even better as they do not introduce any chemicals, et al!
They also do not always require filtering, or it is very simple.
Does it make sense to split 92-95 into 2 points?

101-102 sentence, references are required.

Line 105 “[1-13C]HP001” comes without chemical name

Fig 4, please add values to the figure as it is hard to get the value at this scale.

277 - 278 “Before our arrival at Aarhus University Hospital, a healthy rat had been imaged already by hyperpolarizing HP001 on site and using 278 trityl radicals and a SpinAligner polarizer46,47” This reads oddly as if they could not wait for you. Likely it is historically correct, but maybe rephrase the part 278-290. A suggestion can be that you say that two experiments were performed on the same day, with transported HP001 and on-site polarized…..

398 “in the person of” – maybe better change to a more modern phrase like “…at the University of Eastern Finland by Dr Mikko Kettunen.”

Reviewer #3

(Remarks to the Author)
I would like to thank the authors to carefully revising their manuscript. I believe it reads much better now without any claims/promises that go unfulfilled.

I understand the logistic difficulties of performing the experiment again, and while I still believe that a successful metabolic experiment would make the work much more exciting, I can agree with the other reviewers and return to my original point that the results presented are still very nice.

Since my other comments were addressed by the authors, I am now happy to recommend publication.

Reviewer #4

(Remarks to the Author)
Your manuscript presents a technically impressive and timely demonstration of across-cities transportable ^{13}C hyperpolarization enabled by UV-induced labile radicals, followed by in vivo MRSI in rats. This is a major achievement in the field.

The revised additions have improved scientific clarity, provided necessary mechanistic detail, and strengthened methodological transparency. Most concerns from a first review round appear well addressed.

However, some added text is overly detailed, occasionally disrupts narrative flow, or introduces concepts without adequate framing for a multidisciplinary audience. A few descriptions could be tightened or clarified to avoid interpretive ambiguity, but it doesn't take away from the impressive body of work this manuscript reports on.
Personally, I would try to break down some of the longer sentences in the methodological explanation parts of the manuscript, but this could be a personal writing style preference. Overall, it deters the reader from fully grasping the concepts only a tiny bit.

Open Access This Peer Review File is licensed under a Creative Commons Attribution 4.0 International License, which permits use, sharing, adaptation, distribution and reproduction in any medium or format, as long as you give appropriate credit to the original author(s) and the source, provide a link to the Creative Commons license, and indicate if changes were made.
In cases where reviewers are anonymous, credit should be given to 'Anonymous Referee' and the source.
The images or other third party material in this Peer Review File are included in the article's Creative Commons license, unless indicated otherwise in a credit line to the material. If material is not included in the article's Creative Commons license and your intended use is not permitted by statutory regulation or exceeds the permitted use, you will need to obtain permission directly from the copyright holder.
To view a copy of this license, visit https://creativecommons.org/licenses/by/4.0/

REVIEWER COMMENTS

Reviewer #1 (Remarks to the Author):

This work represents a clear and valuable continuation of the study led by Capozzi and Ardenkjær-Larsen. Building on their previous advancements in small dDNP systems, photo-induced radicals, and hyperpolarization storage units, the authors now demonstrate a proof-of-principle transport of 13C hyperpolarization between cities. Similar hyperpolarization transport experiments have previously been performed with xenon, and the concept of such transportation for 13C-labeled molecules was, among others, discussed by Kempf et al. in JACS (2015).

A particularly notable strength of this study is the integration of multiple technical advancements in hyperpolarization, which together enabled approximately five hours of transport with only ~50% polarization loss—an impressive achievement. It is unfortunate that the concept could not be immediately used for pyruvate-like molecules due to rapid relaxation.

I recommend this work for publication. However, the current version of the figures is of low visual quality, and some font sizes are too small to read comfortably. I hope this will be addressed in the final version. Furthermore, despite the authors' efforts, the manuscript still contains some jargon that is not always self-explanatory, with definitions only found in the Methods section. To improve accessibility for a broader readership, I strongly encourage a critical revision of both the text and the figures.

We thank the reviewer for his/her generally positive comment and willingness to recommend this paper for publication after revision. The quality of all figures has been improved and the text in smaller font size increased and reported in bold. Doing so, we noticed a mistake and replaced the dataset in Figure 1A. Moreover, high definition vectorized figures were submitted together with the revised version of the paper. All jargon and non-self-explanatory issues pointed out by this reviewer have been addressed according to his/her list of comments (see below)

Lastly, the statement: "Raw data are available from the corresponding author (andrea.capozzi@epfl.ch) on reasonable request." is no longer acceptable. Raw data must be deposited in publicly accessible repositories supported by Nature Publishing, national, or European open science initiatives. This practice is now a standard requirement and reflects good scientific conduct.

The data availability statement was modified as "All the raw NMR, ESR, magnetic field and temperature data that support the findings of this study and the MATLAB code to analyse them are available in "Zenodo" with the identifier "doi: https://doi.org/10.5281/zenodo.17465538

Moreover, source data for Figures 1 to 4 are provided with the paper. As an excel file as requested by Nature Comm policy"

Some other comments.

The introduction is nice; however, it does not discuss Xenon hyperpolarization. I see that you are formally right, that here is the first 13C MRI experiment; however, it is not the first transport of hyper in humans in general, and not the longest.

Thanks for pointing it out. Although this work focuses on ^{13}C, it is fair to mention ^{129}Xe transport. In the introduction we added the sentence

"Lifting these restrictions and making 13C hyperpolarization transportable, as already shown for ^{129}Xe on the scale of several hours [31],"

and added reference 31 to the work from Repetto et el. "HP-Xe to go: Storage and transportation of hyperpolarized 129Xenon, https://doi.org/10.1016/j.jmr.2016.02.011"

146-147 "The high field ESR spectrum, measured by means of a LOngitudinal Detection

technique (LOD-ESR)" – It would be helpful if you could explain what this is. You are doing this for the DNP spectrum, and it helps to streamline the text.

We thank the reviewer for the comment. Nevertheless, it is important to notice that in the text we state that "we overlay the LOD-ESR spectrum to the DNP one". We do not use LOD-ESR to measure the DNP spectrum.

Differently from conventional ESR that excites the electron spin in a resonant cavity with a microwave B1 perpendicular to the static field B0, the LOD-ESR technique consists in detecting the electron spins signal on the same axis of the static field B0 using a solenoidal pick-up coil. It is not a resonant technique, and the electron spin excitation is performed by simply switching ON and OFF the microwave output (i.e. pulse modulation). The frequency of the pulse modulation is fed into a lock-in amplifier used to demodulate the signal and obtain the spectrum. The fact that it is not a resonant technique has the advantage to be easily implementable in a dDNP setup with no need for a ESR resonant cavity. The price to pay is low sensitivity, but the latter is compensated by the size of a usual dDNP sample, the high field and very low temperature. Since this paper is a communication, an extensive explanation of this technique is beyond scope. Nevertheless, we added the following sentence and extra reference to address the reader to what we think is a didactical paper on the topic.

"It is important to notice that a LOD-ESR setup is easily implementable on dDNP polarizer with no major hardware modifications. Indeed, the LOD-ESR technique measures the electron spin signal along the same axis of the static field B0 using a solenoidal pickup coil and with no need for a resonance cavity. For further details, see the seminal work by Granwehr et al. [44]"

180-181 "No further decrease was observed for higher values of FM." This statement is not clear to me, as I do see the decline in Figure 1D. Why are the error bars so different for blue and red?

Thanks for pointing this out. The sentence was modified to "No significant decrease was observed for higher values of FM".

The error bars of the relaxation curve (blue) and excitation curve (red) are different because of the LOD-ESR technique. Indeed, when the time constant of the process is close to the physical constant of the pickup coil (approx. 10 ms), the sensitivity of the LOD-ESR

spectrometer increases. This can be seen in Figure S9 where some of the raw date leading to the time constants in Figure 1D were reported as example. The theoretical explanation behind this observation was previously published by our group and reported in references 42 and 43.

Figure 1: Some text elements are too small. The green arrow does not have an immediate visual explanation; maybe you can design it with a thick tail instead of connecting it to the angle.

The font was increased as well as the thickness of the arrow.

202: (panel A) – Figure 2A?, and below for B

Figure 2 was reformatted to have both letters A and B top left.

214 – “the magnetically enforced probe of the polarizer” – please explain the meaning of this

Thanks for pointing this out. Although, to this regard, we clearly reference our previous works (i.e. “Details concerning the design of the magnetically enforced probe of the dDNP polarizer and portable cryostat were described earlier [37,41]”), we recognize that the term is very technical. We explain the concept behind the “magnetically enforced probe” in the previous sentence. Nevertheless, to make it clearer, the sentences were rephrased as follows.

“It is important to notice that different arrangements of permanent magnets constituting a “magnetic rail” were mounted around the dDNP probe and inside the portable cryostat to always keep the sample in a magnetic field of at least 100 mT, along the extraction path from the isocenter of the 6.7 T superconductive magnet to the one of the 1 T permanent storage magnet. Construction details of the permanent magnets arrangements were described earlier [37,41].”

221- “Halbach array along the polarizer probe” – what is the polarizer probe? MW or NMR coil? Does not make sense. And I do not see it on the figure.

Thanks for pointing this out. We apologize for the confusion. The term “polarizer probe” is indeed misleading. The correct one is “dDNP probe”. A brief explanation of what a dDNP probe is was introduced in the part describing Figure 1C:

“Figure 1C shows the ESR and DNP properties of the sample at 1.2 K and 6.7 T. To perform this characterization, a home-built dDNP polarizer was used. The latter included a cold bore superconductive magnet equipped with a cryogenic solid-state NMR probe able to detect the 13C signal as well as to shine microwaves onto the frozen sample by means of a waveguide and a non-resonant cavity (see Methods for details about the dDNP polarizer). From now on, the cryogenic NMR probe with microwave irradiation capability will be referred to as the dDNP probe 35,41.” Moreover, it was marked on Figure 3.

Fig 3: please assign all relevant components: the gate valve, dynamic seal, the magnetically enforced probe, the gate valve of the transportation, the magnetically enforced probe of the portable cryostat, and others

In general although this figure probably adequate it does not help to a reader who did not design the system and not familiar with the jargon used in the lab.

The key elements required by this reviewer were added to the figure; the figure legend was also clarified as follows:

“Sketch of sample extraction and storage step-by-step procedure: step 1 - after thermalization, the sample is manually lifted from the microwave cavity (a), along the dDNP probe (b), up to a removable loading chamber (d); step 2 - the dDNP polarizer’s gate valve (c) is closed and the loading chamber disconnected from the polarizer and docked to the transport cryostat; step 3 - the gate valve of the transport cryostat (e) is opened and the he sample pushed through a magnetic rail (f) down to a liquid He cooled permanent magnet (g), to reach a solenoidal NMR coil (h) (top panel).”

Moreover, to better describe the sketch, the following paragraph was added in the main text:

“The extraction from the dDNP polarizer (Figure 3, top panel) into a purpose-built transportable liquid He cryostat with NMR capability 41 took approximately 50 s and happened in three steps (see Supplementary video 2). Firstly, the sample was raised from the isocenter of the superconductive magnet to the sample loading chamber. Secondly, the dDNP polarizer gate valve was close, the loading chamber with sample inside removed and docked on top of the portable cryostat. Thirdly, the gate valve of the portable cryostat was opened and the sample quickly lowered into the liquid He cold storage permanent magnet.”

317-318 “The achieved level of contrast in the images was not far from what was obtained from a standard trityl radical-doped sample polarized on-site.” – is it reported? Could be reminded here, but it should be at least a factor of 2 different.

Thanks for pointing this out. The angiogram acquired when injecting the contrast agent polarized on site using trityl radical is reported in figure 5 A and B which had indeed a higher snr compared to figure 5 C and D (angiogram acquired with long-lived transported contrast agent). Indeed, the noise starts to appear on frame 8 in Fig 5 C and on frame 15 in Figure 5 A. To avoid any confusion, the concerned sentence was rephrased as:

“Although the angiogram acquired using the transported MCA showed lower SNR, the image contrast was high enough to visualize the same main anatomical element of the rodent”

337 - “Getting rid of methyl group relaxation, via deuteration (i.e. dTMP)” – can you comment on how deuteration helps? I assume relaxation via tunneling should also be in the deuterated case.

The point raised by the reviewer is correct, tunnelling happens also in case of deuteration. Nevertheless, deuteration significantly reduces the rate of quantum mechanical tunnelling in methyl groups because the heavier deuterium atoms make tunnelling through a potential barrier much less probable. This happens because the tunnelling probability is inversely related to the mass of the tunnelling particle. As a result, deuteration slows down or even "quenches" processes like internal rotation of the methyl group.

This was clarified adding the following sentence and reference.

“Indeed, deuteration significantly reduces the rate of quantum mechanical tunnelling in methyl groups because the heavier deuterium atoms make tunnelling through a potential barrier much less probable (Bodi et al, 2015).”

338 – “After thermalization, the abundant nuclear species in our samples (i.e. 1H and 2H) are completely depolarized because of fast spin diffusion during heating” – How? If all nuclei are hyperpolarized, then diffusion should not be so important here unless there is a rapid relaxation on its own.

Spin diffusion is concentration dependent. In the sample 1H/2H are in concentration of 50 M (defined in the text as abundant nuclear species). 13C nuclei are at 2 – 5 M.

404 - HP001 was it also purchased in S-A? Would be helpful to have a catalog numbers for all commertial products that readers can reproduce and have ideas about purity of the used compounds.

Yes, as specified in the methods, “All chemicals were purchased from Sigma-Aldrich (Brøndby, Denmark) excepted for the radical precursor deuterated trimethylpyruvic acid (dTMP) that was synthesized in house, and the AH111501 trityl radical purchased from Polarize ApS (Frederiksberg, Denmark).” Unfortunately, not all products have a catalogue number. Trityl is one of those (see here https://polarize.dk/consumables/). However, providing the correct nomenclature of the product and the retailer company is enough for correct identification of the products included here.

What about trytil? Was it also purchased in S-A?

Thanks for pointing this out. AH111501 trityl radical was purchased from Polarize ApS (Frederiksberg, Denmark). We added this information in the method.

“quartz Dewar (Magnettech” – catalog id?

We are sorry, we could not find a catalogue ID. Moreover, Magnettech has been bought by Bruker. Today, a suitable quartz dewar can be bought here: https://www.bruker.com/en/products-and-solutions/mr/epr-instruments/magnettechesr5000.html.

“The CFP is built using two concentric tubes (an outer lumen - black - and an inner lumen - red -

426 ending with a nozzle - green-)” – Do you refer to any figure? Otherwise, not sure what the colors could mean.

Thanks for pointing this out. We indeed refer to Figure 2. The reference was added in the methods.

Reviewer #2 (Remarks to the Author):

Nature Communications manuscript 25-29843 by Capozzi et al describes the generation of long-lasting 13C hyperpolarization using quenchable UV-induced radicals. The relatively stable 13C hyperpolarization, with T1s in excess of 10 hours, allows for off-site centralized production followed by transportation to and use by a remote study site. A successful demonstration was given for production and utilization between two sites 5 hours and 320 km apart.

As also indicated by the authors themselves, this is highly analogous to the centralized production of 18F-FDG for PET imaging and eliminates, for 13C hyperpolarization, the need for very expensive hyperpolarization equipment. This is a major step forward in the more routine and widespread use of 13C hyperpolarization methodology.

Overall, the work is described clearly and with sufficient detail, such that I can recommend publication with only minor suggestions.

We thank this reviewer for appreciating the quality of our work and his/her overall positive comment. His/her comments improved the quality of the manuscript. Below we address the specific comments.

Specific comments:

1. The text on top of Page 5 in the Introduction is a bit confusing. It is stated that for traditional EPAs "they must be produced on site because of their minute-long half-life in the liquid state". However, this is not really the relevant part as the half-life of any hyperpolarization in the liquid-state is short-lived independent of the radical used. A few sentences later, the authors make the relevant statement themselves stating "they would destroy the MCAs' nuclear spin order within few milliseconds when, during extraction from the polarizer, the frozen HP sample is exposed to low magnetic field." I think this entire section can be rephrased more clearly, stating the relevant considerations for both traditional and UV-induced radicals.

Thanks for pointing this out. To avoid any confusion, we rephrased the section as follows:

"A main drawback is that, after dissolution, all HP 13C MCAs are short-lived (i.e. minute-long half-life of the HP state).

Although, the half-life of HP 13C MCAs is orders of magnitude longer when frozen and kept at cryogenic temperatures[29,30], samples prepared with traditional EPAs, such as trityl, cannot be extracted as a frozen solid from the dDNP polarizer without losing their HP state. Indeed, paramagnetic relaxation at low magnetic field (i.e. when moving the sample far from the magnet's isocenter) would destroy the high nuclear spin order within few milliseconds[31].

Currently, should one be willing to equip an MRI facility with hyperpolarization, the only way is to place, as close as possible to the scanner, costly and technically demanding hardware (i.e. the dDNP polarizer). Lifting these restrictions and making 13C hyperpolarization transportable, as already shown for 129Xe on the scale of several hours[32], would allow

centralized production of hyperpolarized substrates followed by transport to multiple locations for HP MRI use, in a similar fashion as for 18F-fluorodeoxyglucose (18F-FDG) PET tracers, dispensing with the requirement to have a dDNP polarizer in close proximity[13]."

2. Page 15 – For consistency with earlier text in the Introduction, use MRSI instead of CSI. There is no need to use 2 acronyms for the same technique.

done

3. Figure 5 - This Figure can benefit from text within the panels. Instead of fully relying on text within the legend, add labels like 'trityl-DNP' and 'UV-DNP'. Add time stamps to the dynamic series. Generally, it is good to be able to understand the content of a figure without having to read the legend.

Thanks, very good suggestions. We implemented them all and modified the legend accordingly

4. Page 21, line 393 - … it was earlier demonstrated that changing …

done

Reviewer #3 (Remarks to the Author):

I started reading the paper with great interest as transportation of HP 13C agents would be a real game-changer for the field, and for the application of the technique in clinical practice. Hence, I was a little disappointed seeing that only perfusion HP agent was successfully used (with 50% polarization, loss). While this is still a very nice result, HP 13C will not make it to clinical use thanks to perfusion scans. There are plenty of other options already available for that. It is the metabolic imaging possibilities of HP 13C (that the authors describe in the rather long introduction) that are of real clinical interest. Unfortunately, the metabolic imaging experiment tried in this study did not work. To achieve a real impact, this needs to change.

We thank this reviewer for the time spent on reading and critically commenting on our work and his/her general interest in the idea of making hyperpolarization transportable. We also understand his/her disappointment in not seeing metabolism in this study. Nevertheless, we find that his/her comments are criticizing the paper for something it was never claimed to achieve. The core critique - that the manuscript lacks a successful metabolic imaging demonstration - misrepresents the intended scope of our work.

The study focus is on the technical feasibility of transporting hyperpolarized agents over long distances, which we have carefully and transparently framed. While we find the broader potential of this capability genuinely exciting, we have deliberately avoided overinterpreting our findings.

We made a concerted effort throughout the manuscript to avoid overstating the significance of our findings, and we explicitly discussed all key limitations in the discussion. This level of transparency, in our view, underscores the scientific rigor of our approach rather than an attempt to "oversell" the results.

Regarding the use of a clinical scanner, we made no claims about clinical readiness or immediate translation. What was available in Aarhus was a clinical scanner: the use of a clinical scanner simply reflects the available infrastructure at that site. We injected a rat, and for sure we cannot inject a human - neither now, nor next year. We clearly state that this

work remains preclinical, and that regulatory aspects such as ethics approvals for human studies and safety validations are beyond the current scope.

We recognize that an *in-vivo* metabolic study after transport would represent a very bold result. Unfortunately, at present, performing new imaging experiments in Aarhus is not feasible due to current limitations in instrumentation availability. The experiment involved substantial coordination across institutions and logistical challenges, particularly given the long-distance transport from Copenhagen to Aarhus. Despite these constraints, we successfully transported and imaged two distinct substrates: HP001 (the first perfusion agent studied with hyperpolarization) and glucose, a substrate that has potential to open the application span of hyperpolarized MRI if a solution can be found to delivery challenges given by its short liquid state T1. Hyperpolarized perfusion agents have just made it to the clinic: a recent demonstration of clinical brain perfusion was carried out at UCSF (https://www.nature.com/articles/s44303-025-00073-3). Demonstrating that a perfusion agent can be transported over such a long distance and still yield imaging results comparable to those obtained with on-site polarization represents quite an achievement, as underlined by the other reviewers.

At the same time, we acknowledge that the introduction stresses too much clinical use of hyperpolarization and metabolism. Although our intent was to introduce the potential of HP MRI in general to a non-expert reader, we recognize that this could be misleading for the message held by rest of the paper. For this reason, we changed this part of the introduction focusing on the challenge of obtaining long-lived hyperpolarized species rather than on the clinical applications and metabolism. Also, we modified the Conclusions stressing the current limitations of the technique. Finally, any clinical claim was removed from the Abstract. (see revised version of the paper).

Main points:

This work is an extension of the authors work in transporting UV polarized agents (refs 36 and 41) with the use of new transport device (ref 40) to allow “across-cities” transport. The distance over which the polarization was transported being the main (and likely only) novelty. As stated above, this could still be considered exciting, should you be able to demonstrate a successful metabolic experiment. Please consider making the trip again to deliver this essential piece of data.

The reviewer characterizes the transport distance as the sole novelty, but overlooks 3 main findings of our work:

1- The fact that the molecular environment of the target molecule plays a crucial role in terms of solid-state spin-lattice relaxation parameters is a key factor in transport success. Initially, we thought that the only caveat was the presence/absence of the radical in the sample. This insight carries significant implications for future development of transportable hyperpolarized agents. This observation was only possible by performing a thorough 13C relaxometry investigation of several samples. Ref 40 (the design of the transportable cryostat) did not report any 13C experiment.

2- Ref 40, did not describe any HP sample extraction experiment. In this work we demonstrated that the permanent magnets arrangements, basically allow a loss free

extraction procedure. As underlined by reviewer 2 in his/her additional comment, demonstration of the complete workflow is crucial in this kind of experiments.

3- This work reports the first in-vivo preclinical HP MRI experiment using a transported HP contrast agent. HP imaging needs high signal, and this is the demonstration of the fact that our workflow is robust.

Concerning extra experiments, we are sorry but at the moment we are not able to perform them because of infrastructure reorganization issues. We are in the process of moving this research elsewhere.

The fact that you have used a “clinical” scanner is hardly of importance given you are still scanning a rat using a substrate not allowed for clinical use. Please don’t try to oversell the work.

Overselling was not our intention. In the text, we removed any claim about the use of a clinical scanner. The GE 3 T scanner is mentioned only in the Methods.

You explain that error in corrected T1s came from calibration of B1, while there is definitely an effect, it is more likely that you are not able to properly fit such long T1s with your current experiment (only up to 7h readout). Also, your claim that everything is fine up to a non-corrected T1 below 10h, does not hold as your experiments shown that dTMP with 10.3h T1 still has a negligible error. Please don’t make up cut-offs without any evidence.

We acknowledge the confusion of our statement. The B1 correction data was removed from Fig 4 and we simply mention the T1 value that could be observed. The paragraph was rephrased as follows.

“The glucose ^{13}C T_1 of the reference sample at 4.2 K and 1 T measured 0.87±0.01 hours for trimethylpyruvic acid (TMP) as radical precursor and in a protonated solvent, 10.3±0.1 hours for d_4-trimethylpyruvic acid (dTMP) as radical precursor and in a protonated solvent, 9.9±0.1 hours for AKG as radical precursor and in a protonated solvent, and 17.7±0.2 hours for AKG as radical precursor and in a deuterated solvent. Figure S5 in Supporting Information shows the decay of each sample during the liquid He holding time of the transportable cryostat (7 h).”

In the discussion section, you try to balance out the fact that you lost 50% polarization during transport by promoting UV induced polarization without persistent radicals in the solution. While this is indeed a nice feature of these solutions, the benefits of this polarization technique has been discussed elsewhere, Plus it only makes your case worse as what you are saying basically is “If we made these on site we could have skipped the QC and injected faster”, hence loosing even less polarization in comparison to the transported sample. Or do you want to say that your sample had to go through QC procedure at the recipient site, which was probably not necessary and could have saved you some polarization? If yes, state that rather than general stories. Also, would it be possible to use polarization by UV induced radicals for both transported and non-transported samples?

We acknowledge the confusion and accept the criticism in particular “in vision of a clinical use”. We rephrased based simply on what was observed in this study and mentioned that the same sample produced over 50% liquid state polarization when polarized on site.

“Although the angiogram acquired using the transported MCA showed lower SNR, the image contrast was high enough to visualize the same main anatomical element of the rodent. Half of the polarization was lost during the full experimental procedure and 5 h transport. Indeed, it was earlier demonstrated that, hyperpolarizing HP001 using UV-radicals at 6.7 T/1.2 K and dissolving it on-site generated close to 50% 13C polarization in the liquid state[33]”

Talking about stories, the whole manuscript reads more like an adventure story than a scientific work, please consider rewriting.

We acknowledge that the part of the manuscript describing the dissolution off-site shows the problem pointed out by this reviewer. We rewrote as follows:

“The sample formulation providing the longest relaxation time (2 M glucose and 0.7 M AKG dissolved in d8-glycerol:D2O) was used to perform HP MRSI in vivo. The experiment took place at the MR Research Center of Aarhus University Hospital (Aarhus, Denmark), where we transported a long-lived HP sample from the Technical University of Denmark (Kgs, Lyngby, Denmark). The transport took 5 h and covered 320 km (see Supplementary video 3). Once on site, we performed the dissolution from the transportable cryostat and injected a rat through its tail vein, previously prepared in a 3 T scanner, with 1 mL of HP glucose solution at 60 mM (see Supplementary video 4).

MRSI was acquired with the observation frequency centered on the c2-c5 glucose multiplet. Considering losses during transport, an elapsed time of 30 s between dissolution and beginning of the MR acquisition, and a glucose liquid-state T1 of 15 s36, we estimated a 13C polarization between 5 and 7 % at time of injection. As a first attempt and because of the broad chemical shift of uniformly 13C,d7 labelled glucose the entire HP signal was used to acquire a single image (see Figure S7 in Supporting Information).”

One minor point. For better comparison between the two perfusion experiments, I would recommend dropping the first “pre-perfusion” frame.

Done. Figure 5 was updated.

Reviewer #4 (Remarks to the Author):

Overall the authors have made an interesting and innovative contribution to the field of MRSI. The methodology does feature some cumbersome elements, such as the lengthy transportation, but it is a step towards democratisation of MRSI and they do contribute to increased sensitivity. Overall, the manuscript does contribute to the scientific body of literature and is an adequate proof of concept work.

We thank de reviewer for his/her generally positive comment. We would just like to specify that, among other reason related to the readiness of the HP MRI platform for the planned experiment, we chose to travel from Copenhagen to Aarhus (lengthy transportation) to prove the robustness of our method and test our long-lived HP compounds on a trip of over 5h.

Minor comments attached to the documents as comments.

We extracted the comments from the attached files:

1- In the legend of Figure 3 of the main manuscript, the reviewer asked to mention/credit the software used to generate the images reported in the top panel. The software used to generate the figures is SolidWorks 2023 (Dassault Systèmes, Vélizy-Villacoublay, France,). This information was added to the methods in the subsection “Processing, data analysis and CAD”:

“All CAD drawings related to this project as well as the technical images present in Figure 2 and Figure 3 were realized using SolidWorks 2023 (Dassault Systèmes, Vélizy-Villacoublay, France,)”

2- In the method part the reviewer asked to provide more details about the “home built dDNP polarizer”. In the original version of the paper we mention reference 18 because the system used was conceptually similar to the one invented and used in the seminal work by Ardenkjaer-Larsen et al in 2003. The description after reference 18 is actually a synthetic description of the main elements of the polarizer. Nevertheless, we added 2 more references and the following sentence because over the years the system was improved and retrofitted.

“All DNP and LOD-ESR were performed on a homebuilt dDNP polarizer (6.7 T magnet from Magnex, Oxford, UK) working at 1.15±0.05 K conceptually similar to the idea introduced in 2003 [18]. The original system (3.35 T) was retrofitted to work at higher field and employ the CFP technology [41,42]. Indeed, the polarizer was equipped with a 94 GHz solid-state source (Polarize Aps, Copenhagen, Denmark) coupled to a 200×2R4 frequency doubler (VDI, Charlottesville, VA, USA), which provided an output power of 100 mW at 188 GHz. The source, digitally controlled through NI-DAQ device USB-6525 (National Instruments, Austin, TX, USA) had a tuning range of ±1 GHz and the possibility to modulate the output frequency at a rate up to 10 kHz and with an amplitude of up to 500 MHz. All 13C NMR acquisitions were performed using a Varian INOVA console (Palo Alto, CA, USA) connected to a low temperature probe equipped with permanent magnets along the central loading tube 37, placed inside the polarizer VTI (Variable Temperature Insert), remotely tuned to 71.8 MHz.

3- In the Methods part in the “Magnetic field measurements and simulations” the reviewer asked to share the MATLAB code to generate the magnetic field profile of all components used int this study. The code was shared in the repository mentioned at the beginning of this document.

4- In the legend of Figure S7 (panel B) in supporting information, the reviewer asked to add the chemical shift axis and assign peaks. We are sorry but we cannot fulfil this request. This is beyond our capability and the scope of the manuscript. Each frame (a voxel of the MR image) of panel B shows the full spectrum, so a 50 ppm chemical shift scale should be assigned to each of them. Moreover, the acquisition was performed in vivo and on a 3 T scanner. The SNR and shim level do not allow us to distinguish the different peaks beyond the “binary” observation “in this voxel we have signal” vs “in this voxel we have no signal”. As a comparison, we attach here the spectrum recorded on a high-resolution magnet (9.4T) and in vitro for the sample HP sample polarized on site. The spectrum clearly shows all 6 carbons (c1 to c6) of the glucose molecule.

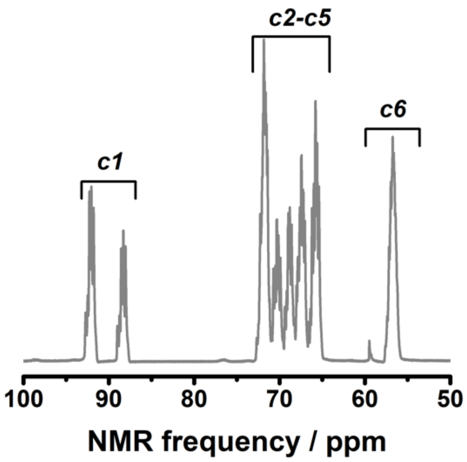

-------Additional comments of reviewer #2 ---------------------------------------

I read the comments from Reviewer 3, and I do agree with them to a certain extent.

We thank this reviewer for the extra-time dedicated

The statement that 'the metabolic imaging experiment tried in this study did not work' is not entirely accurate as the paper does demonstrate a 5-7% polarization for glucose. However, I do agree that glucose is an unfortunate substrate that will likely never yield impressive metabolic images from downstream metabolites. This is because the polarization of glucose disappears quicker than the metabolic processes it undergoes (glycolysis). For this reason, the 13C HP community has been using HP pyruvate as this substrate bypasses glycolysis and measures a very fast reaction (pyruvate <> lactate). A demonstration of 'across-cities' studies for HP pyruvate would greatly strengthen the manuscript.

We thank this reviewer for the extra time dedicated to our work and the attention paid to all details.

The work is largely based on two aspects, (1) UV polarized agents and (2) a novel transportation device. These two aspects have been described separately, whereby the current manuscript is a combination and extension of the previous work. Personally, I do not have a problem with this as science is an incremental process whereby small aspects are generally described separately. The current work is still a strong cumulative conclusion of the earlier work.

Thanks for stressing this point. This study is indeed the first complete demonstration that hyperpolarization can be produced off-site, transported and generate HP imaging in vivo.

Overall, I stand by my earlier recommendation. However, I do feel that a demonstration for HP pyruvate would strengthen the work.

Thanks. We are working on it at a collaborator site (Dr Mikko Kettunen, University of Eastern Finland). Unfortunately, the departure of the PI of this project from DTU makes new experiments very difficult at the moment.

REVIEWER COMMENTS

Reviewer #1 (Remarks to the Author):

This is very nice proof-of-concept work that fits the journal perfectly. I recommend it with minor corrections for publications.

96-97 lines read as if point 1 (line 89) has an advantage over point 2 (line 92) because it is readily biocompatible. It feels that polymers could be even better as they do not introduce any chemicals, et al!
They also do not always require filtering, or it is very simple.
Does it make sense to split 92-95 into 2 points?

Lines 92-95 were split in 2 points to separate the case of polymers (more biocompatible) from powders. The paragraph now reads:

"So far, to circumvent this shortcoming, research has pursued two different approaches:

- To use UV-induced labile radicals that can be quenched, after the DNP process has happened in the solid state inside the polarizer, by heating up the sample above 190–200 K by means of a thermalization process[33–36];
- To physically separate carbon nuclei from the EPAs by utilizing differences in solubility of radicals and 13C-MCAs[38];
- To physically separate carbon nuclei from EPAs by grafting the radicals inside purpose synthesized porous polymers that can absorb a solution containing the substrate of interest39.

HP substrates polarized with UV-induced labile radicals, as well as the porous polymers, can have the advantage of being readily biocompatible…"

101-102 sentence, references are required.
references 30, 31, 33 (already present in the manuscript) were added at the end of the sentence

Line 105 "[1-13C]HP001" comes without chemical name
the chamical name bis-1,1-(hydroxymethyl)-[1-^{13}C]cyclopropane-d_8 was added after [1-13C]HP001

Fig 4, please add values to the figure as it is hard to get the value at this scale.

Done, Fig 4 was updated reporting numerical values close to the data points.

277 - 278 "Before our arrival at Aarhus University Hospital, a healthy rat had been imaged already by hyperpolarizing HP001 on site and using 278 trityl radicals and a SpinAligner polarizer46,47" This reads oddly as if they could not wait for you. Likely it is historically correct, but maybe rephrase the part 278-290. A suggestion can be that you say that two experiments were performed on the same day, with transported HP001 and on-site polarized…..

We implemented the suggestion. Thanks. The paragraph now reads:

“Two experiments were performed the same day on two healthy rats. The first rat was imaged by hyperpolarizing HP001 on site and using trityl radicals and a SpinAligner polarizer[42,43]. The liquid-state signal of a 150 mM single peak 13C solution was used to acquire a complete angiogram by means of a gradient echo sequence with frames every 0.5 s. Figure 5A shows the rat cardiovascular system’s highlight starting from the tail vein injection. Complete coverage of heart and kidneys was achieved at frame #8 (Figure 5B), after 4 s from injection. Afterwards, the signal slowly started to fade out.

The hyperpolarized angiogram was repeated on a second rat using the transported hyperpolarized signal (Figure 5C).”

398 “in the person of” – maybe better change to a more modern phrase like “…at the University of Eastern Finland by Dr Mikko Kettunen.”

Thanks for the suggestion. Now the paragraph reads:

“The latter would allow shorter (i.e. within the same city) transport, and it is, at the moment, object of investigation at the University of Eastern Finland by Dr Mikko Kettunen.”
